# Off-On–Off Cascade Recognition of Cyanide, Mercury, and Aluminum Using *N*/5-Monosubstituted Rhodanines

**DOI:** 10.1021/acsomega.4c01066

**Published:** 2024-04-04

**Authors:** Sinan Bayindir, Abdullah Saleh Hussein

**Affiliations:** † Department of Chemistry, Faculty of Sciences and Arts, Bingol University, Bingol 12000, Türkiye; ‡ Department of Chemistry, Graduate School of Natural and Applied Sciences, Bingol University, Bingol 12000, Türkiye

## Abstract

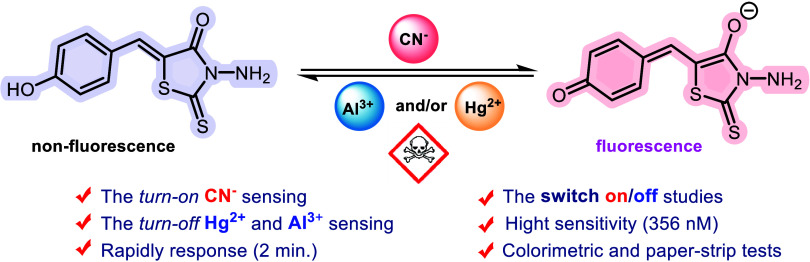

This study aims to synthesize *N-* and 5-monosubstituted rhodanine derivatives as ion-sensing organics and investigate their sensing abilities. Following an easy and green approach to synthesis, the anion-sensing properties of the rhodanines were studied using colorimetric detection and spectroscopic methods. As a result of studies, rhodanines are found to be highly solvent-controlled colorimetric and fluorescent cyanide, mercury, and aluminum sensors. The stoichiometry of the interaction between CN^−^ and both probes was determined to be 1:1 using Job’s plot analysis. The binding constants (*K*_s_) of CN^−^ to **5-arylRh** and ***N*****-arylRh** were calculated to be 3.25 × 10^4^ and 7.07 × 10^4^ M^–1^, respectively, demonstrating their high affinity for cyanide ions. The limits of detections for the **5-arylRh** and ***N*****-arylRh** were also determined as 356 and 617 nM, respectively. In addition to detecting CN^−^, **5-arylRh** also serves as a specific turn-off sensor for mercury and aluminum when cyanide and hydroxide are present. This enables the fluorescence intensity to be toggled on/off by alternating the addition of CN^−^/OH^−^ and Hg^2+^/Al^3+^. Furthermore, the LOD values for Hg^2+^ and Al^3+^ with **5-arylRh**–CN^−^ and **5-arylRh**–OH^−^ were determined to be 414 nM and 1.35 μM, respectively. Furthermore, the turn-on binding mechanisms of **5-arylRh** and ***N*****-arylRh** with cyanide ions were elucidated, and the experimental band gap (highest occupied molecular orbital/least unoccupied molecular orbital) energy values corroborated the proposed mechanism. Additionally, the interaction mechanism of the probes with CN^−^ was further investigated by using the ^1^H NMR technique. Collectively, these findings suggest that **5-arylRh**, ***N*****-arylRh**, and **5-arylRh**–CN^−^ hold promise as selective and sensitive candidate sensors for CN^−^, Hg^2+^, and Al^3+^ ions.

## 1 Introduction

Ions make up a crucial class of chemical substances that play an essential role in many physical, chemical, and biological processes. The recognition of anions is therefore essential for research in medicine, industry, biology, and environmental science.^1−5^ Cyanide ions are the most acutely toxic inorganic anions. Cyanide is fatally harmful to humans and other ecosystems, so it must be carefully monitored.^6^ Cyanide ions are persistently generated in the environment through industrial processes such as plastic production, electroplating, metallurgy, and leather tanning. Moreover, industrial activities, such as organic reagent manufacturing, photography, and mining, release large amounts of cyanide into the environment.^7,8^ Cyanide is a highly toxic chemical that can kill quickly, especially in mammals like humans. It affects the respiratory, cardiovascular, and nervous systems. Exposure to even a small amount of cyanide for just a few minutes can be fatal.^9,10^ On the other hand, the Environmental Protection Agency (EPA) has set the maximum contamination level for cyanide ions in drinking water at 1 μM.^11^ The World Health Organization (WHO) has set the highest tolerable dose of cyanide ions in drinking water at about 2 μM.^12^ These low levels have led scientists to develop precise methods for detecting and destroying this harmful pollutant. Cyanide ions have a strong attraction to metal ions, forming complexes that are less toxic. This property is used in some wastewater treatment processes to remove cyanide from industrial effluents. However, this is both more expensive and can lead to the formation of different toxic substances, although relatively less.^13,14^ Therefore, it may be more beneficial to use organic-based binders for the determination and removal of cyanide ions.^15^ Hence, cyanide ion detection is important for human health care and environmental protection. It is typically based on hydrogen bonding or deprotonation of CH, NH, and OH groups, which changes the optical properties of colorimetric or fluorescent organic compounds.^16^ There are many methods for cyanide ion recognition based on various mechanisms, such as deprotonation-protonation and complexation with a probe through hydrogen bond formation.^17^ In these methods, the deprotonation of an acidic proton present in the receptor ensures a low detection limit, high specificity, and selectivity.^18−20^ In addition, while the benefits of mercury and aluminum reach deep into industries and daily life, both pose significant threats to our health and the environment. Mercury, a potent toxin second only to plutonium, can contaminate our air and water from industrial activities.^21,22^ Even minute amounts of Hg^2+^ in drinking water can harm living beings, sparking global efforts to purify water and curb its release.^23^ Yet, currently, available detection methods are costly and cumbersome, hindering our ability to track and manage this threat effectively.^24^ Similarly, aluminum, though useful in food additives and water treatment, has a harmful side.^25^ Its soluble form wreaks havoc on ecosystems, while excess accumulation within us can trigger neurodegenerative diseases and disrupt vital bodily functions.^26,27^ Though safety guidelines exist, the sheer pervasiveness of aluminum necessitates constant vigilance.^28^ To navigate this complex landscape, we must understand the delicate balance between the benefits and risks. To safeguard health, meticulous monitoring of Hg^2+^ at all concentrations (ppb, ppm, and μM) is vital, while WHO suggests a daily aluminum intake of 3–10 mg/kg and a 7.41 μM Al^3+^ limit in drinking water.^12,29,30^ In this context, organic-based sensor candidates are increasingly popular, and many researchers are designing organic-based probes.^31^ The key is to ensure that these organics are both practical, straightforward, and cost-effective, with their design being fully optimized.^32^ Within this framework, exploring the anion-sensing capabilities of organics containing acidic/basic or electrophilic/nucleophilic groups, along with hetero atoms facilitating robust hydrogen bonding, is of interest and remarkable area.^33,34^ Moreover, determining the affinity of organics with different combinations of the same nature toward ions is important in this context. In studies conducted in recent years, rhodanine derivatives, a class of heterocyclic compounds, have been reported to have also remarkable ion-sensing properties in addition to their medicinal properties.^35,36^ Among these derivatives, 3-amino-rhodanine (**1**, 3-NH_2_–Rh) has recently attracted significant attention from researchers. Some studies have been conducted on the efficient synthesis and potential applications of *N*- or C5-substituted derivatives of 3-NH_2_–Rh.^37−40^ Inspired by these studies, we synthesized *N*/C5-monosubstituted rhodanine derivatives with identical side groups. We then investigated the anion-sensing properties of these ligands by using colorimetric and spectroscopic techniques.

## 2 Results and Discussion

### 2.1 Chemistry

**Scheme 1:**
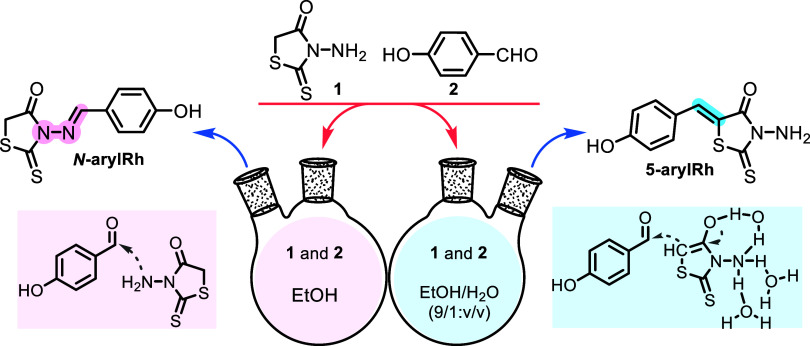
Synthesis Strategy of ***N*-arylRh** and **5-arylRh**

Organic sensor candidates are increasingly popular, and many researchers are designing organic-based probes. It is important that these organics are practical, simple, and inexpensive and that their design is optimized. Determining the affinity of organics with different combinations of the same nature toward ions is also important in this context. In this regard, rhodanine, a heterocyclic compound with a rich chemical structure, is a promising material for ion detection studies. The first part of this work involves synthesizing simple rhodanine derivatives, ***N*****-arylRh** and **5-arylRh**, which contain identical groups at different positions. The target organics were prepared simply by a water-promoted regioselective reaction approach without any catalyst, following a procedure outlined in the literature.^40^ The first part of this study involves synthesizing rhodanine derivatives ***N*****-arylRh** and **5-arylRh** (Schemes 1 and S1). This was done following a procedure outlined in the literature. The synthesis of the first rhodanine derivative, ***N*****-arylRh**, is a typical ylide reaction that was obtained by reacting 4-hydroxybenzaldehyde (**2**) and 3-NH_2_–Rh (**1**) in ethanol at room temperature. The synthesis of **5-arylRh** was carried out using a water-supported Knoevenagel condensation reaction of 4-hydroxybenzaldehyde (**2**) with 3-NH_2_–Rh (**1**). This contrasts with the typical reactions of amines and aldehydes.

### 2.2 Spectroscopic Characteristics

**Figure 1:**
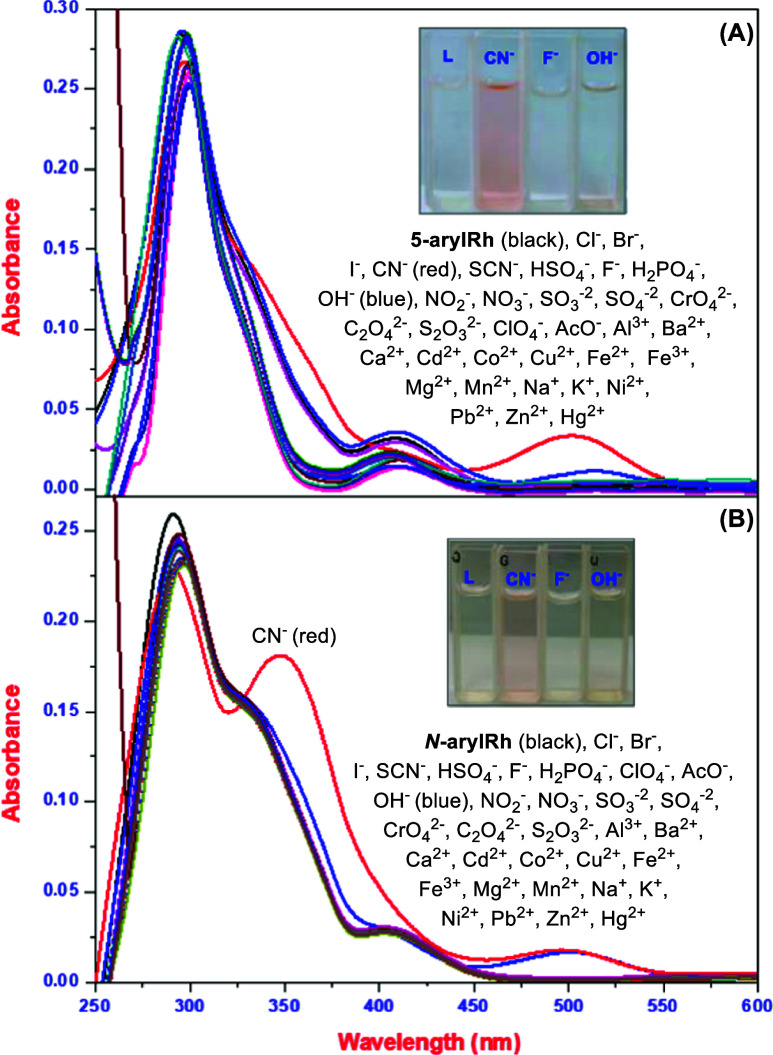
UV–vis spectra of **5-arylRh** (A) and ***N*-arylRh** (B) in THF/H_2_O with various ions; inset: colorimetric responses of Rhs with selected anions.

The simple rhodanines ***N*****-arylRh** and **5-arylRh** contain many hydrogen bond donors and acceptors and acidic protons. As a result, we investigated the anion-detection properties of rhodanines ***N*****-arylRh** and **5-arylRh** in the second part of the study. Following synthesis, the rhodanine derivatives ***N*****-arylRh** and **5-arylRh** were studied for their interactions with a wide range of cations (Al^3+^, Ba^2+^, Ca^2+^, Cd^2+^, Co^2+^, Cu^2+^, Fe^2+^, Fe^3+^, Hg^2+^, K^+^, Mg^2+^, Mn^2+^, Na^+^, Ni^2+^, Pb^2+^, and Zn^2+^ as their chloride salts) and anions ([Bu_4_N]F, [Bu_4_N]Cl, [Bu_4_N]Br, [Bu_4_N]I, [Bu_4_N]AcO, [Bu_4_N]HSO_4_, [Bu_4_N]ClO_4_, [Bu_4_N]CN, [Bu_4_N]SCN, [Bu_4_N]H_2_PO_4_, [Bu_4_N]OH, NaNO_2_, NaNO_3_, Na_2_SO_4_, Na_2_SO_3_, Na_2_C_2_O_4_, Na_2_CrO_4_, Na_2_S_2_O_3_) in 2-propanol, acetone, *N*,*N*-dimethylformamide (DMF), dimethyl sulfoxide (DMSO), ethanol (EtOH), methanol (MeOH), tetrahydrofuran (THF), dichloromethane (DCM), toluene, acetonitrile (CH_3_CN), and their aqua solvent systems. The solvation studies of Rhs in these solutions revealed no major changes in either their color or their absorption properties (Figure S3A). On the other hand, Rhs exhibited strong interactions with cyanide and fluoride ions in aprotic solvents (DMF, acetone, THF, CH_3_CN, DMSO, and their low water ratio) depending on the water ratio and time, but not in protic solvents (2-propanol, MeOH, EtOH, and H_2_O) (Figure S3A–B). Despite similar behaviors in aprotic solvents, the strongest interactions of ligands were observed in THF. For these reasons, THF and THF/H_2_O were chosen as the most suitable solvent systems for the studies. UV–vis absorption studies of **5-arylRh** and ***N*****-arylRh** (10 μM) in THF and THF/H_2_O at room temperature were performed to investigate the interaction of new rhodanine derivatives with anions (Figure 1A–D). Rhodanines **5-arylRh** and ***N*****-arylRh** in THF changed color from colorless to red when exposed to cyanide, fluoride, and hydroxide anions (3 equiv). Fluoride ions rarely interact with organics in aqueous solutions. Therefore, studies were performed in THF with an increasing water content (Figure S3E,F). As expected from studies with **5-arylRh** at an increasing water content (Figure 1B), **5-arylRh** did not interact with fluoride. Unlike **5-arylRh**, ***N*****-arylRh** interacted with hydroxide and cyanide in a THF/H_2_O (v/v: 9:1) solution but did not interact with any ions at higher water ratios (Figure 1D). In summary, both ***N*****-arylRh** and **5-arylRh** interacted with cyanide, hydroxide, and fluoride ions in pure THF, but only **5-arylRh** interacted specifically with cyanide ions in increasing water ratios. Considering all of the initial optimization investigations, we established our operating conditions as THF and THF/H_2_O (v/v: 8:2) for **5-arylRh**, and solely THF for ***N*****-arylRh**. To comprehensively explore the interaction of novel rhodanine derivatives with anions, we conducted ultraviolet–visible (UV–vis) absorption studies on **5-arylRh** and ***N*****-arylRh** (each at a concentration of 10 μM). These experiments were performed at room temperature in both THF and THF/H_2_O (v/v: 8:2). Subsequently, absorption spectra for **5-arylRh** and ***N*****-arylRh** in the presence of various anions were recorded within 1–10 min after the addition of 10 equiv of each respective ion. In this context, when we looked at the absorbance spectra of **5-arylRh** and ***N*****-arylRh** derivatives in THF and THF/H_2_O, we saw that they had similar spectral behaviors. This means that the absorption spectra of **5-arylRh** and ***N*****-arylRh** showed typical rhodanine and heteroarene absorption bands, with strong bands at 295/290 nm for **5-arylRh** (Figure 1A,B) and 296/290 nm for ***N*****-arylRh** (Figure 1C,D). These observations may imply that the (S=C–N–C=O) bonds, attributed to the conjugation of the aryl group with (HN–C=O) bonds, result in a shift of n−π* and π–π* transitions toward longer wavelengths.^35,36^ Subsequently, we investigated how the **5-arylRh** and ***N*****-arylRh** derivatives (10 μM) interact with a variety of ions (30 μM). The studies were conducted in THF, and they showed that **5-arylRh** interacts with fluoride, cyanide, and hydroxide ions. These interactions caused the characteristic ligand absorbance band at 295 nm to red-shift to around 305 nm in the presence of the cyanide, hydroxide, and fluoride ions (Figure 1A). Additionally, new interaction bands emerged at approximately 358–365 and 516–518 nm. Similar patterns were observed in the case of ***N*****-arylRh**, albeit with minor variations, when it interacted with cyanide, hydroxide, and fluoride ions (Figure 1C). As mentioned earlier, we showed that the properties of **5-arylRh** and ***N*****-arylRh** ligands change depending on the amount of water in a THF/H_2_O mixture. In the initial optimization experiments conducted with **5-arylRh** in THF/H_2_O (v/v: 8:2) and ***N*****-arylRh** in THF/H_2_O (v/v: 9:1), the ligands exhibited notably strong characteristic features at around 295 nm, and it was observed that they produced a distinct absorbance peak. In the investigations involving **5-arylRh** in THF/H_2_O (v/v: 8:2), a notable interaction absorbance peak at 515 nm was solely observed in response to cyanide ions (Figure 1B). On the other hand, when we studied ***N*****-arylRh** in THF/H_2_O (v/v: 9:1), interaction peaks were identified at approximately 498 nm for both cyanide and hydroxide ions (Figure 1D). In summary, these remarkable findings demonstrate that both **5-arylRh** and ***N*****-arylRh** interact with cyanide, hydroxide, and fluoride ions in THF and in THF/H_2_O (v/v: 9:1). In addition, **5-arylRh** specifically interacts with cyanide ions in THF/H_2_O (v/v: from 8:2 to 6:4) solvent systems.

**Figure 2:**
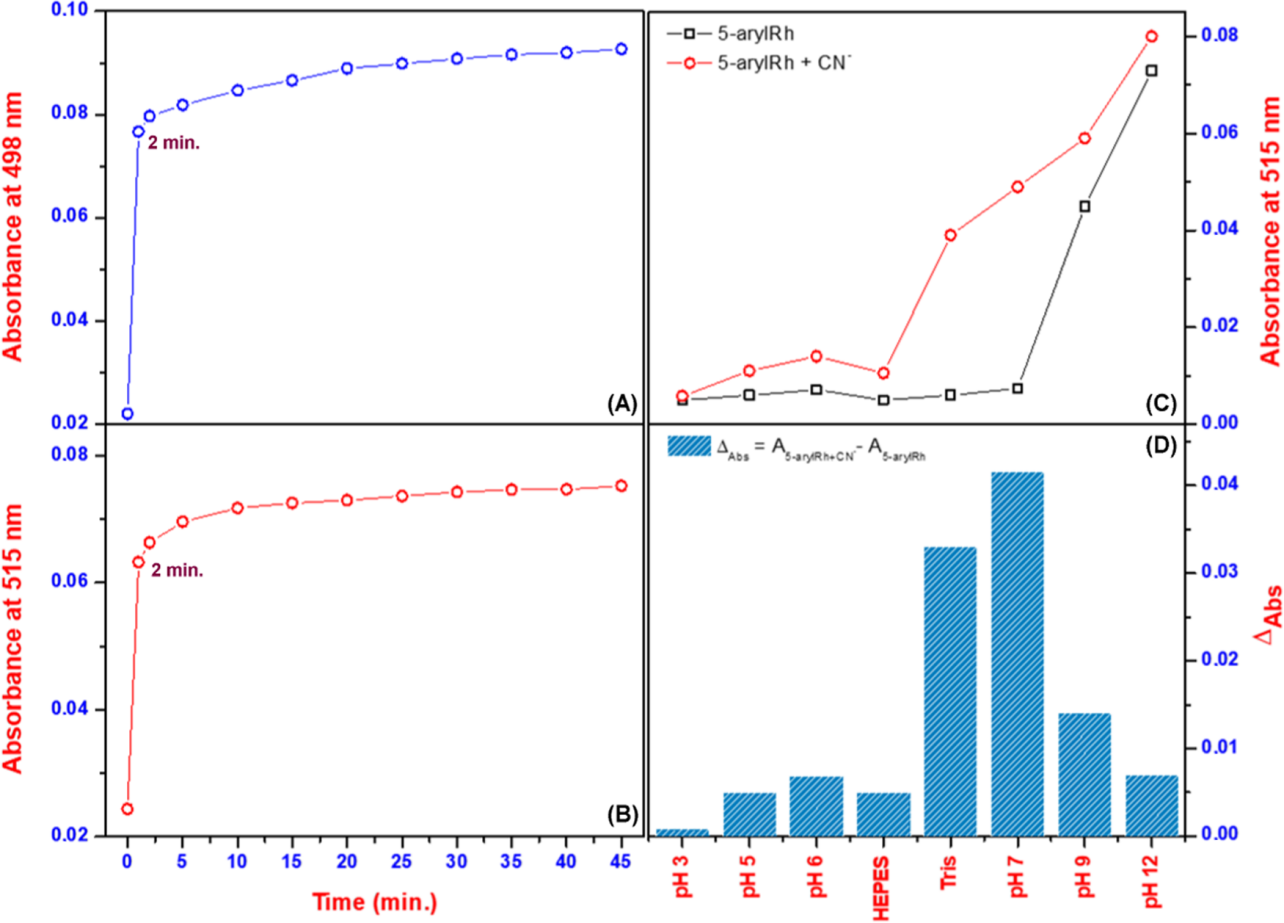
Exposure times of the ***N*-arylRh** (A)/**5-arylRh** (B) with [Bu_4_N]CN, and the absorbance values of **5-arylRh** with [Bu_4_N]CN at different pH (3–12) values (C, D).

Following general UV–vis studies, in this section, to further investigate the anion-sensing properties of **5-arylRh** and ***N*****-arylRh**, the absorbance of probes exposed to the ions was monitored over time. Figure 2A,B shows that exposing the probes to cyanide ions at room temperature caused a dramatic increase in the initial absorbance intensity of **5-arylRh**/***N*****-arylRh** at 515:498 nm, indicating strong binding interactions between **5-arylRh**/***N*****-arylRh** and cyanide ions. Remarkably, Figure 2A,B shows that the absorbance of **5-arylRh**/***N*****-arylRh** at 515:498 nm increased by more than 50% after just 2 min of exposure to cyanide ions. Additionally, the absorbance of ***N*****-arylRh**/***5*****-arylRh** at 498:515 nm increased by more than 50% after just 2 min of exposure. This analogous trend was also observed in the case of fluoride and hydroxide ions in THF (Figure S4). These results suggest the potential of these uncomplicated probes for the swift detection of cyanide. Similarly, pH studies are also important to characterize sensor candidate organic probes (Figure 2C,D). Because the ***N*****-arylRh** derivative does not interact in aqueous environments, we studied the pH studies of **5-arylRh**, which can be studied in aqueous environments. We adjusted the pH of the environment using NaOH and HCl. The detailed procedure is given in the Supporting Data. First, we studied **5-arylRh** at different pH values (Figures 2C and S5). Notably, it was observed that **5-arylRh** lacks any discernible absorbance peak between pH 2 and pH 7. However, a distinct absorbance peak emerged at approximately 515 nm, accompanied by a color change from colorless to red, under basic conditions (from pH 8 to pH 12). We observed similar behavior in the presence of [Bu_4_N]OH in THF, but a very weak activity peak in THF/H_2_O with an increasing water ratio. These results show that **5-arylRh** interacts independently of cyanide ions under basic conditions (from pH 8 to pH 12). Under similar conditions, cyanide ions did not interact with **5-arylRh** at acidic pH values (2–6), but they did interact strongly under basic conditions. However, the difference in absorbance (Δ_Abs_) values of **5-arylRh** and **5-arylRh**–CN^−^ changed very little under basic conditions (Figure 2D). Finally, we studied the interaction of **5-arylRh** with cyanide ions at pH 7 and observed a stable peak that could be attributed to only cyanide ions. Additional studies were also conducted using different buffer systems to explore the observed effect. While THF/HEPES (v/v: 8:2) proved unsuitable for obtaining conclusive results, experiments in THF/Tris (v/v: 8:2) successfully replicated the earlier findings. This suggests that the Tris buffer and neutral pH might be essential for the observed effect. Overview, these sensor candidates excel in specific pH environments, but their versatility is limited by their inability to operate across a wider pH range. That is, the interaction peak at 515 nm also arose in the presence of hydroxide ions within the Tris buffer. Additionally, these simple sensor candidates, despite their rapid response in kinetic studies, exhibit poor performance in aqueous environments and lack specific CN^−^ interactions under HEPES buffer and pH (2–6 and 8–12) conditions.

**Figure 3:**
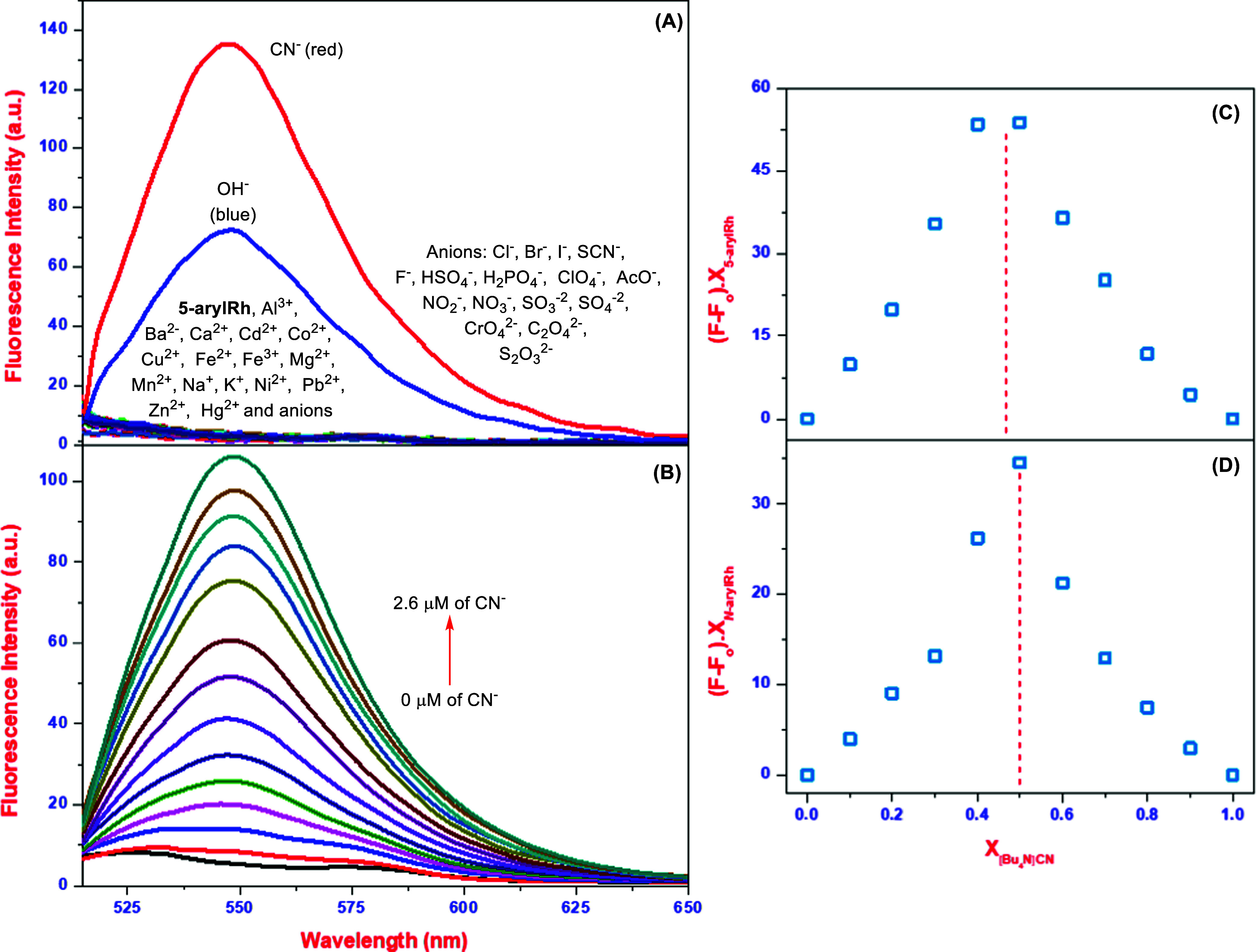
Fluorescence spectra of **5-arylRh** (A) with various ions; (B) fluorescence titration spectra of **5-arylRh** in the presence of increasing [Bu_4_N]CN, and Job’s plot of **5-arylRh** (C)/***N*-arylRh** (D) with [Bu_4_N]CN.

Apart from UV–vis and kinetic investigations, fluorescence spectroscopy experiments were conducted to assess the fluorescence ion-sensing capabilities of **5-arylRh** and ***N*****-arylRh**. These experiments aimed to provide deeper insights into the specific and responsive binding affinity of **5-arylRh** and ***N*****-arylRh** toward a range of chosen anions and cations. As will be remembered, solvent studies of **5-arylRh** and ***N*****-arylRh** showed that both probes interacted with cyanide, hydroxide, and fluoride ions in THF (Figure 1A,C). However, in THF/H_2_O (v/v: 8:2), **5-arylRh** showed a significant interaction band only with CN^−^ ions with a considerably weaker interaction observed with OH̅ ions (Figure 1B). Unlike **5-arylRh**, ***N*****-arylRh** gave relatively lower interaction peaks to cyanide and hydroxide ions than in THF (Figure 1D). Up on this, fluorescence studies were achieved by monitoring alterations in their fluorescence emission spectra in THF and/or THF/H_2_O (v/v, from 9:1 to 5:5). As depicted in Figures 3A and S6, when **5-arylRh** and ***N*****-arylRh** were individually excited at 500 nm, no emission bands were detected for either probe. However, upon introducing ions into the **5-arylRh** solution in THF/H_2_O (v/v: 8:2), a significant increase in emission intensity was only observed in the presence of cyanide, and hydroxide ions, resulting in an emission band at around 547 nm (Figure 3A). Comparable findings were achieved in the studies of the interaction between **5-arylRh** and ions, which were conducted while the water content. Likewise, when ions were added to a solution of ***N*****-arylRh** in THF, the emission band intensity increased significantly in the presence of cyanide, hydroxide, and fluoride ions at around 548 nm (Figure S6C). However, no interaction was observed in the fluorescence studies of ***N*****-arylRh** with ions in THF/H_2_O (v/v, from 9:1 to 5:5) solution. Recalling the absorbance investigations of **5-arylRh** and ***N*****-arylRh** within THF, it was evident that distinctive interaction peaks emerged at around 400 nm. These interactions of **5-arylRh** and ***N*****-arylRh** with ions in THF were explored by using fluorescence spectroscopy with 400 nm excitation. As a result of these studies, the outcome of these investigations revealed that both probes emitted fluorescence exclusively in the presence of hydroxide ions (Figure S6B). Rhodanines, ***N*****-arylRh** and **5-arylRh**, were observed to interact with hydroxide and cyanide ions in fluorescence studies despite the weak ligand interactions observed with hydroxide ions in UV–vis studies in aqueous environments. However, because hydroxide is often used as an indicator of basicity, the quantitative analysis of cyanide ions constitutes the main theme of this study. In this particular, in addition to investigating the spectral properties of the probes toward ions, the fluorescence titration and Job’s plot studies were conducted to calculate limit of detection (LOD), limit of quantitation (LOQ), and *K*_s_ values for the binding of **5-arylRh** and ***N*****-arylRh** to CN^−^ ions. The fluorescence titration experiments were also aimed at elucidating the binding behavior of **5-arylRh** and ***N*****-arylRh** with CN^−^ ions. For this purpose, fluorescence titration experiments were performed by gradually adding [Bu_4_N]CN to **5-arylRh** (10 μM) and ***N*****-arylRh** (10 μM) in THF/H_2_O (v/v: 8:2) and THF, respectively (Figures 3B and S6D). Experiments in the presence of [Bu_4_N]CN showed that the fluorescence peaks at 548 nm for **5-arylRh** and 549 nm for ***N*****-arylRh** increased as the concentration of [Bu_4_N]CN increased. The increase in the peak intensity began at a [Bu_4_N]CN concentration of about 0.2 μM and reached completion at a concentration of 2.6 μM. These fluctuations are attributed to the potential interaction of cyanide with both **5-arylRh** and ***N*****-arylRh**. Following fluorescence titration studies, to determine the binding stoichiometry between **5-arylRh** or ***N*****-arylRh** and CN^−^, Job’s plot experiments were performed as described in the Experimental Section. The fluorescence intensity of mixtures of CN^−^ and **5-arylRh** or ***N*****-arylRh** in varying molar ratios was measured at room temperature. The results showed that **5-arylRh** and ***N*****-arylRh** interact with CN^−^ in a 1:1 ratio (Figures 3C,D and S7).

**Figure 4:**
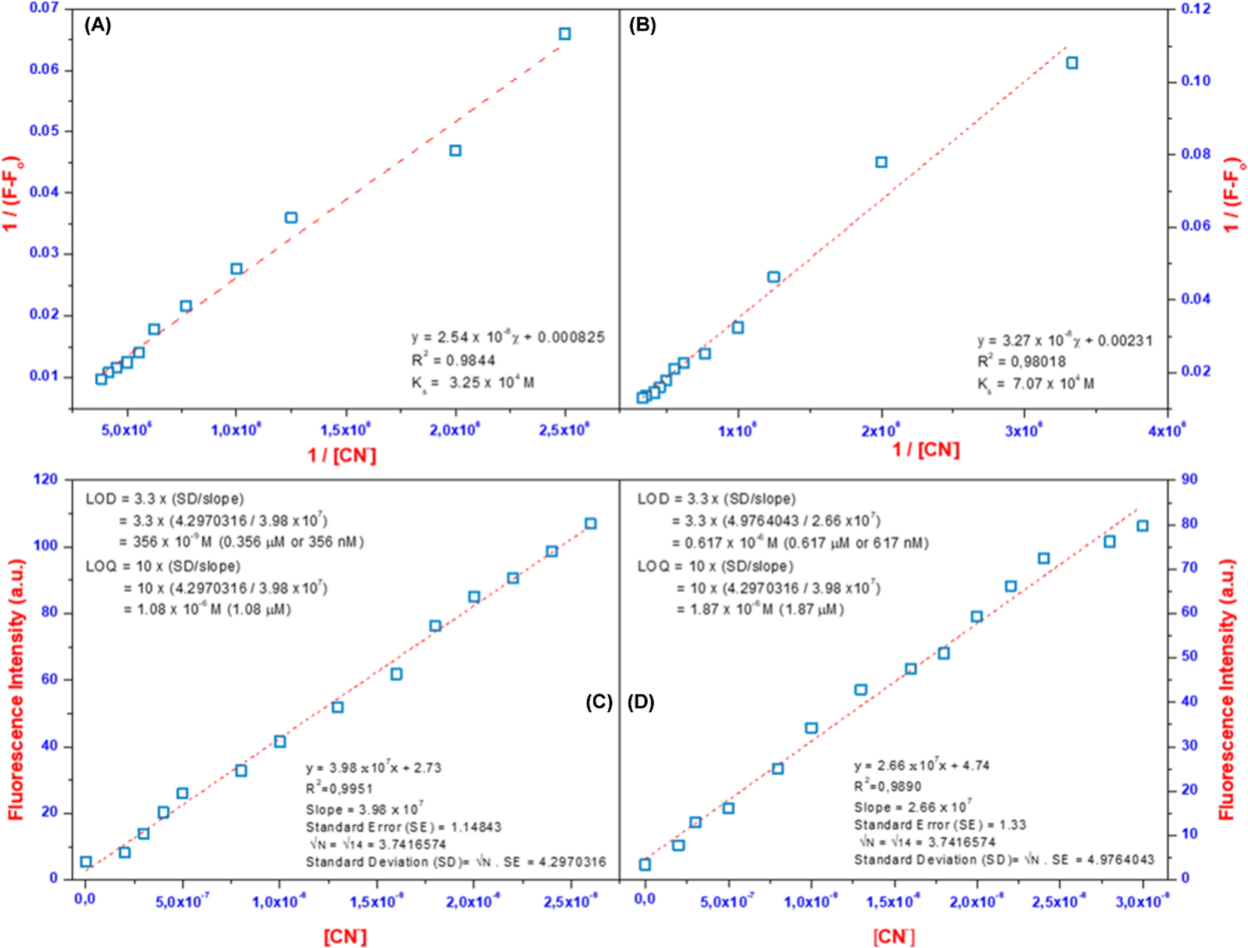
Benesi–Hildebrand plot based on a 1:1 association stoichiometry between **5-arylRh** (A) or ***N*-arylRh** (B) and the change in absorbance intensity of **5-arylRh** (C) or ***N*-arylRh** (D) with the increasing concentration of [Bu_4_N]CN.

After fluorescence titration and Job’s plot measurement, the binding constant (*K*_s_), LOD, and LOQ values of the **5-arylRh** and ***N*****-arylRh** complexes with CN^−^ ions were calculated using fluorescence titration data and the corresponding equations. Initially, the *K*_s_ values of the **5-arylRh** and ***N*****-arylRh** complexes with cyanide ions were obtained from the slope of the graph drawn using the data obtained from fluorescence titration using Benesi–Hildebrand eq 1. The *K*_s_ values of **5-arylRh** and ***N*****-arylRh** with cyanide ions were calculated to be 3.25 × 10^4^ and 7.07 × 10^4^ M^–1^, respectively (Figures 4A,B and S8A,B).
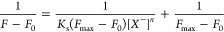
1Subsequently, the LOD and LOQ values were determined for **5-arylRh** and ***N*-arylRh** using fluorescence titration data and the corresponding eqs 2 and 3. Accordingly, the LOD and LOQ values of **5-arylRh** and ***N*-arylRh** were calculated as 356/617 nM and 1.08/1.87 μM, respectively (Figures 4C,D and S8C,D).
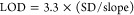
2
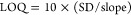
3

### 2.3 Interference and Reversibility Studies

**Figure 5:**
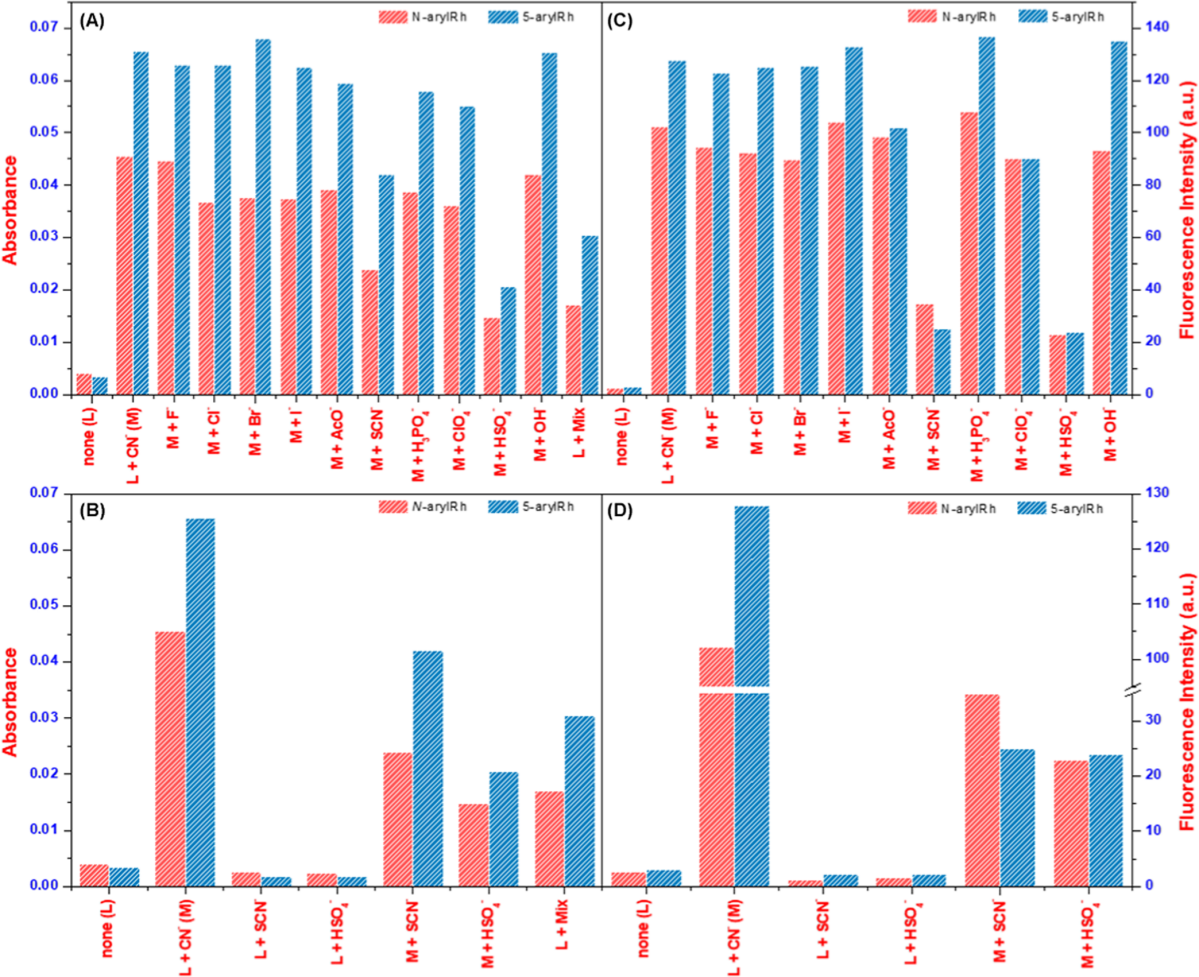
UV–vis (A, C) and fluorescence (B, D) selectivity of ***N*-arylRh**/**5-arylRh** for cyanide in the presence of other anions.

Besides spectroscopic characteristics experiments, analyzing the interference interactions of probes with ions and the corresponding spectral changes is essential. This is because real-world samples rarely contain a single ion, making it necessary to consider the effects of multiple ions on probe interactions. In this context, after UV–vis and fluorescence experiments, an investigation into the impact of various anions on the binding of cyanide ions with ***N*****-arylRh**/**5-arylRh** was undertaken. Competitive experiments involving cyanide and other anions were conducted in THF and THF/H_2_O (v/v: 8:2) solutions (Figures 5 and S9). Building upon the results of UV–vis and fluorescence experiments, we further investigated the impact of competing anions on the binding of CN^−^ ions to ***N*****-arylRh**/**5-arylRh** complexes (Figure 5A,C). Experiments revealed that the increase in absorbance and fluorescence induced by a mixture of CN^−^ with other anions was similar to that caused by cyanide alone in THF and THF/H_2_O (v/v: 8:2). However, in the presence of SCN^−^ and HSO_4_^–^ ions, while relative decreases were observed, no colorimetric change occurred. This indicates that the sensor candidates, **5-arylRh** and ***N*****-arylRh**, do not exhibit colorimetric sensor properties in the presence of SCN^−^ and HSO_4_^–^ ions. Therefore, based on these studies, none of the tested anions interfered with the interaction between ***N*****-arylRh**/**5-arylRh** and cyanide ions (Figure 5B,D). However, the inability of **5-arylRh** and ***N*****-arylRh** to function colorimetrically in the presence of SCN^−^ and HSO_4_^–^ ions, along with minor decreases in UV–vis and fluorescence interactions, can be considered a minor drawback for this study.

**Figure 6:**
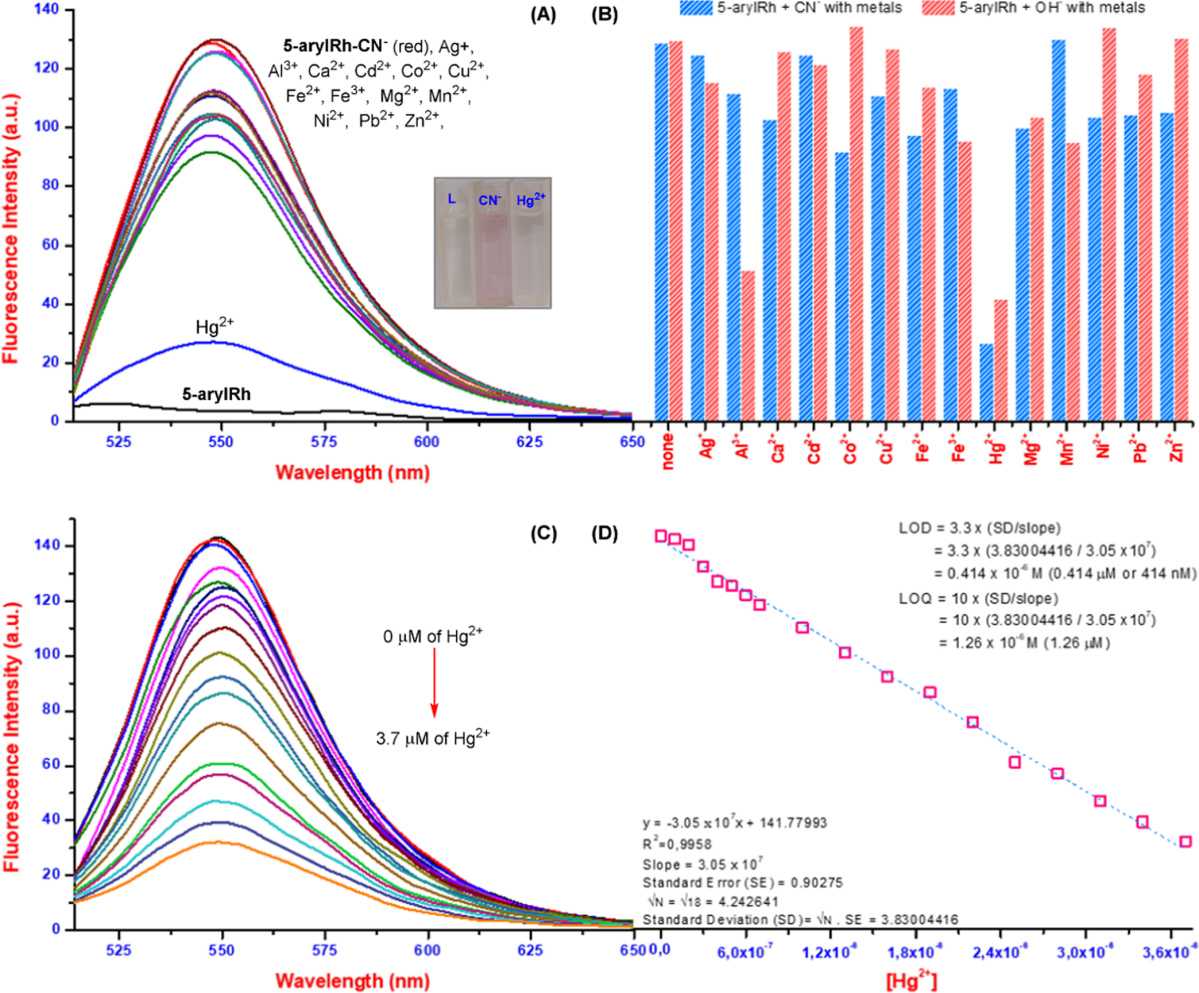
Analysis of **5-arylRh**–CN^−^ Interactions with metals: (A) fluorescence spectra, (B) bar graph, (C) fluorescence titration spectra in the presence of Hg^2+^, (D) fluorescence intensity changes with increasing Hg^2+^ concentration; inset: color changes of **5-arylRh** upon alternate addition of CN^−^ and Hg^2+^ for one cycle.

Additionally, as shown in Figures 1 and 3, absorbance and fluorescence studies of the **5-arylRh** and ***N*****-arylRh** with anions and cations were carried out, and it was determined that the probes did not interact with metals. Next, fluorescence spectroscopy was used to study the interactions of **5-arylRh**–CN^−^ and **5-arylRh**–OH^−^ with metals (Figures 6A,B and S10A). First, the interactions of the **5-arylRh**–CN^−^ probe with metals were examined, and it was found that the fluorescence intensity of **5-arylRh**–CN^−^ at 548 nm decreased approximately 7-fold in the presence of mercury ions. This result suggests that **5-arylRh**–CN^−^ can be used as a specific turn-off mercury sensor.^18^ Subsequently, the interactions of **5-arylRh**–OH^−^ with metals were examined, and it was seen that the interaction peak of **5-arylRh**–OH^−^ at 550 nm decreased not only in the presence of mercury ions but also approximately 3-fold in the presence of aluminum ions. Moreover, the study extended to examine the interactions of the **5-arylRh**–OH^−^–CN^−^ mixture with metals, revealing a reduction in fluorescence intensity by roughly 8- and 4-fold in the presence of mercury and aluminum ions, respectively (Figure S10B). These results imply that **5-arylRh**–CN^−^ can be used as a specific mercury sensor and as a sensor for both mercury and aluminum ions when hydroxide ions are present. Following general fluorescence studies with cations, the sensitivity of **5-arylRh**–CN^−^ and **5-arylRh**–OH^−^ toward Hg^2+^ and Al^3+^ ions, respectively, was investigated using fluorescence titration in THF/H_2_O (8:2 v/v). Both complexes displayed fluorescence quenching upon the addition of the target cations. For **5-arylRh**–CN^−^, fluorescence decreased by about 85% with Hg^2+^, reaching a plateau around 3.7 μM HgCl_2_ (Figure 6C). The LOD and LOQ for Hg^2+^ were 0.414 μM (414 nM) and 1.26 μM, respectively (Figure 6D). Similarly, **5-arylRh**–OH^−^ exhibited sensitivity toward Al^3+^, with a fluorescence difference plateauing around 4.0 μM AlCl_3_ (Figure S10C). The LOD and LOQ for Al^3+^ were 1.35 and 4.09 μM, respectively (Figure S10D). These results demonstrate the potential of both complexes for selective cation sensing. One potential challenge of this study is identifying aluminum and mercury within a mixture. By analyzing the pH of the metal mixture at a neutral level (pH 7), we can gain clues about its composition. In other words, at pH 7, where there are no hydroxide ions present, the interaction with **5-arylRh**–CN^−^ seems specific to mercury ions. On the other hand, if adding hydroxide ions to the mixture at this pH level results in no change, but a spectral change occurs upon the addition of hydroxide, this suggests the presence of aluminum. Summarily, the spectral changes of **5-arylRh**–CN^−^ indicate the presence of mercury ions. In contrast, the presence of aluminum ions is indicated by the spectral changes of **5-arylRh**–CN^−^–OH^−^ or **5-arylRh**–OH^−^.

**Figure 7:**
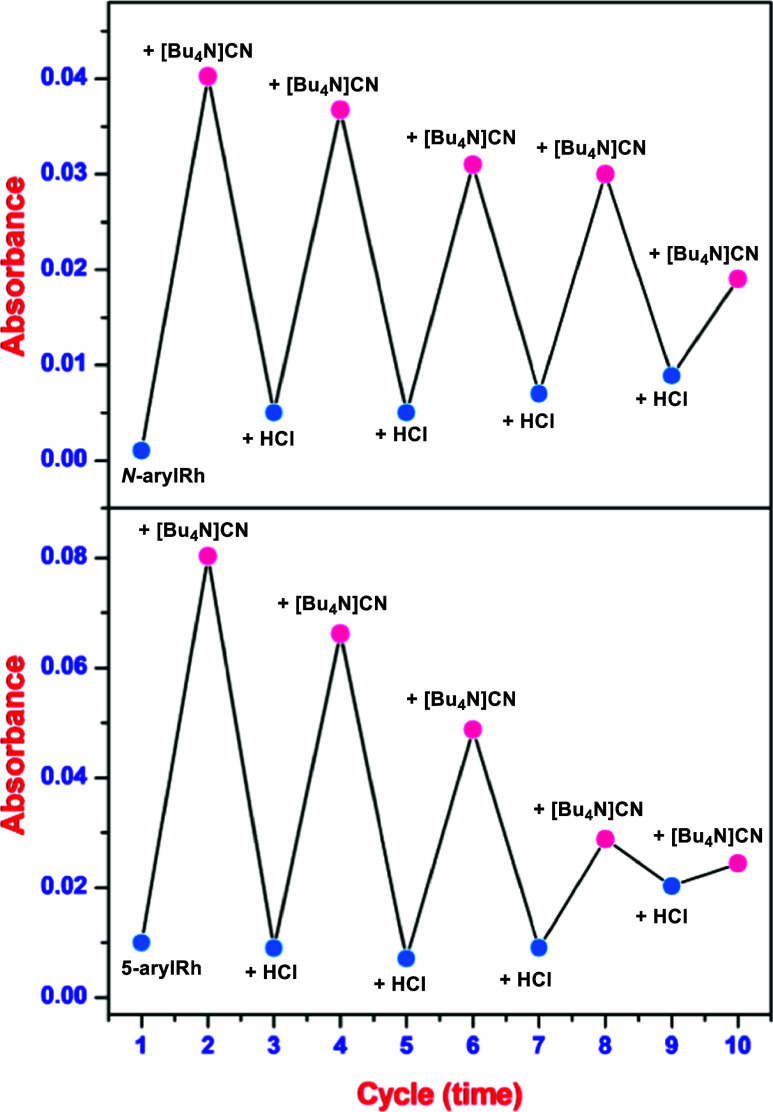
Reversible switching of the absorbance intensity of ***N*-arylRh**/**5-arylRh**.

On the other hand, a notable characteristic of organic probes is their ability to exhibit switchable or reversible sensing properties. In this investigation, the introduction of [Bu_4_N]CN and HCl or trifluoroacetic acid (TFA) alternatively to ***N*****-arylRh/5-arylRh** leads to a toggling on/off change in the absorbance intensity at 515 nm (Figures 7 and S11A,B).^15^ Moreover, switchable on/off variations of **5-arylRh**–CN^−^ were also observed in the presence of Hg^2+^ ions (Figure S11C). However, compared with HCl or TFA, it exhibits an ineffective reversible feature with only three conversions. These studies have demonstrated that ***N*****-arylRh**/**5-arylRh** can be readily reused for CN^−^ and HCl sensing for approximately nine and seven cycles, respectively. Furthermore, a molecular logic function was established based on the optical response of ***N*****-arylRh**/**5-arylRh** to cyanide and HCl as input signals. In this context, an “on” state corresponded to OUTPUT logic 1, while an “off” state corresponded to OUTPUT logic 0, based on the absorbance levels. With this in mind, when the data was configured, the ***N*****-arylRh**/**5-arylRh** chemosensor remained in the “off” state in the absence of the INPUTS CN^−^ (In1) and HCl (In2). Upon the introduction of CN^−^ (In1) to the ***N*****-arylRh**/**5-arylRh**, a noticeable increase in absorbance intensity at 515 nm was observed, leading to an output logic of 1, indicating the “on” state. Conversely, when only HCl (In2) ions were introduced to ***N*****-arylRh**/**5-arylRh**, a reduction in absorbance at 515 nm was observed, resulting in an output logic of 0 (Figure S11D).

Researchers are interested in the detection capabilities of readily available organic compounds with simple structures that they can synthesize. In this context, extensive research has revealed *N*/5-monosubstituted rhodanines as turn-on sensors for cyanide ions in neutral and Tris buffer solutions. Remarkably, these probes also exhibit specific turn-off responses toward Hg^2+^ and Al^3+^ ions in the presence of CN^−^ and OH^−^ ions, respectively. A comparison of the performance of these probes with previously reported CN^−^, Hg^2+^, and Al^3+^ sensors in terms of their *K*_s_ and LOD values is presented in Table 1. As evident from Table 1, the LOD values obtained for the rhodanines are comparable to those reported for CN^−^, Hg^2+^, and Al^3+^ sensors in the current literature, indicating their potential as acceptable cyanide, mercury, and aluminum ion detection agents.^41−49^

**Table 1:**
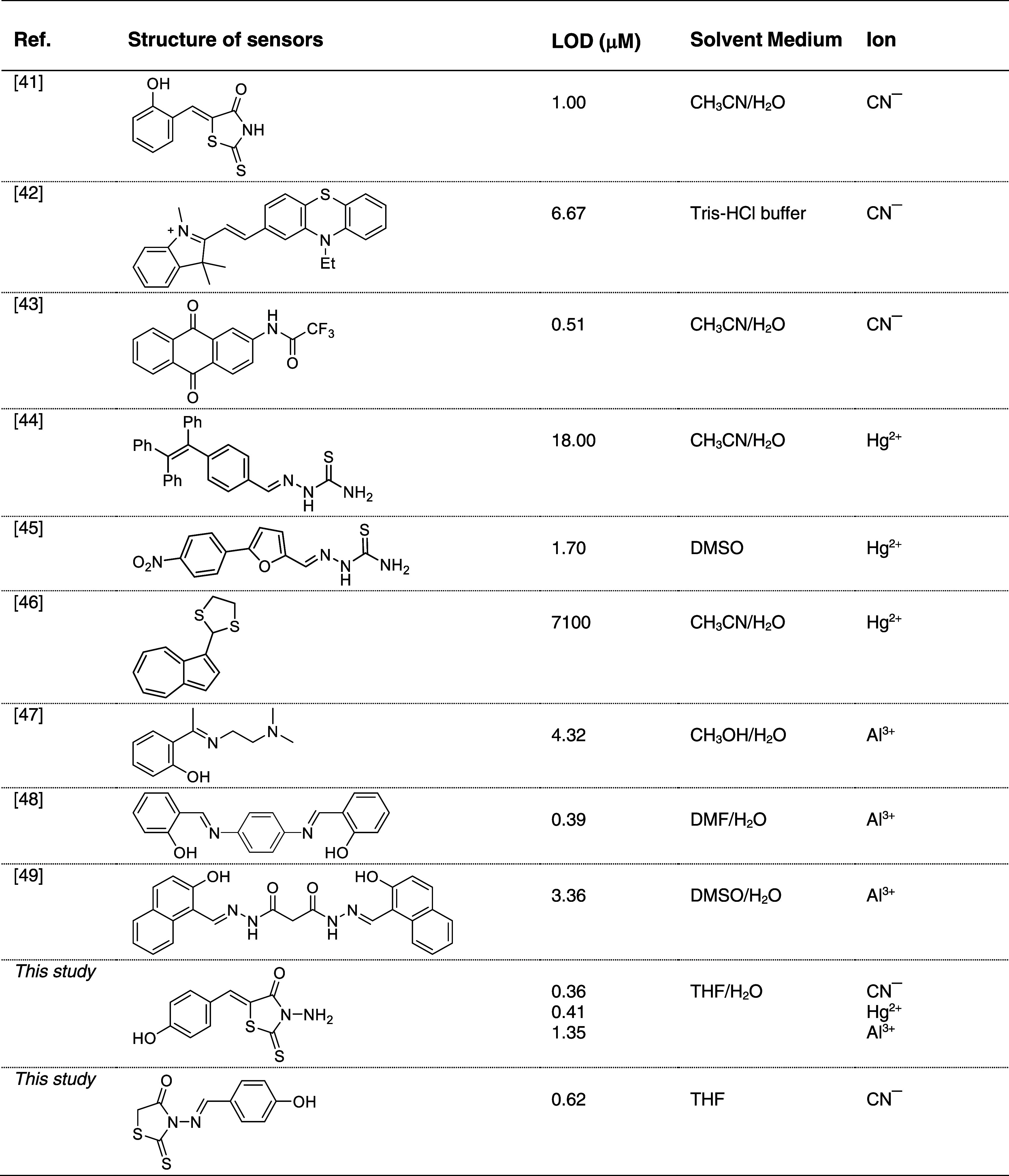
Comparison of Some CN^−^, Hg^2+^, and Al^3+^ Selective Chemosensors

### 2.4 Binding Mechanism

**Figure 8:**
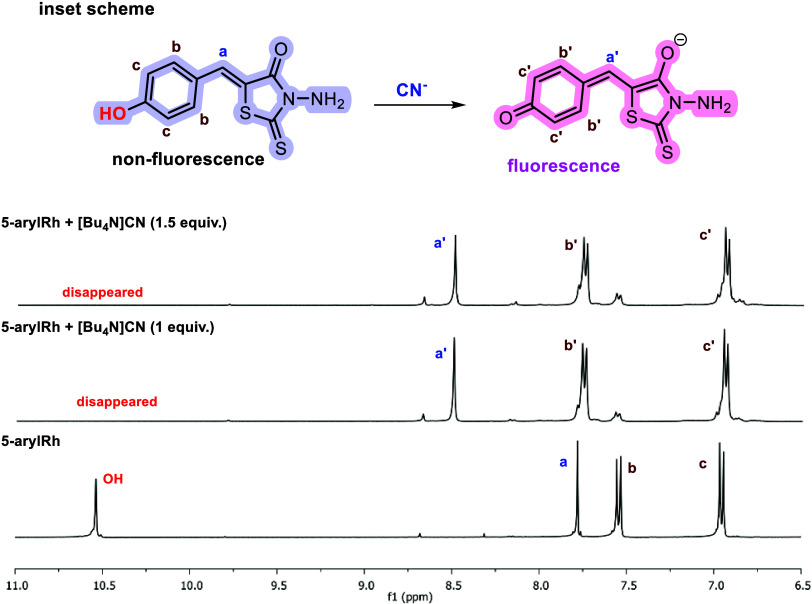
^1^H NMR spectrum of **5-arylRh**, and **5-arylRh** + [Bu_4_N]CN in DMSO-*d*_6_; inset scheme: mechanism of **5-arylRh** for cyanide detection.

Organic Rhs contain functional groups capable of interacting with cyanide ions through nucleophilic, basic, and hydrogen-bonding mechanisms.^10,15,16,36^ This interaction was readily observed by a color change and confirmed by spectroscopic methods such as UV–vis and fluorescence spectroscopy. Furthermore, ^1^H NMR studies in DMSO-*d*_6_ with and without [Bu_4_N]CN provided insights into the binding properties (Figures 8 and S12). An interaction with cyanide ions caused significant shifts in the ^1^H NMR spectrum, revealing information about the binding mode and confirming the observed color change. Given the basic and hydrogen-bonding nature of cyanide, these shifts can be attributed to hydrogen bonding between the acidic OH group of Rhs and the cyanide ions. Over time, proton removal from the cyanide’s hydroxide group leads to the disappearance of OH peaks and the emergence of new peaks associated with the quinone ring and =CH groups. These observations suggest that complexation with cyanide ions impacts the hydroxide protons of Rhs. Additionally, NMR spectra and band gap data indicate that cyanide ions remove a proton from the phenol ring’s OH group, forming a quinone unit. This disrupts the probe’s conjugated push–pull system, weakening the intramolecular charge transfer (ICT) process. Conversely, it strengthens the excited-state intramolecular proton transfer (ESIPT) effect. These combined effects lead to a rise in fluorescence intensity (Figure S13).^1,50,51^

### 2.5 Colorimetric and Paper-Strip Tests

**Figure 9:**
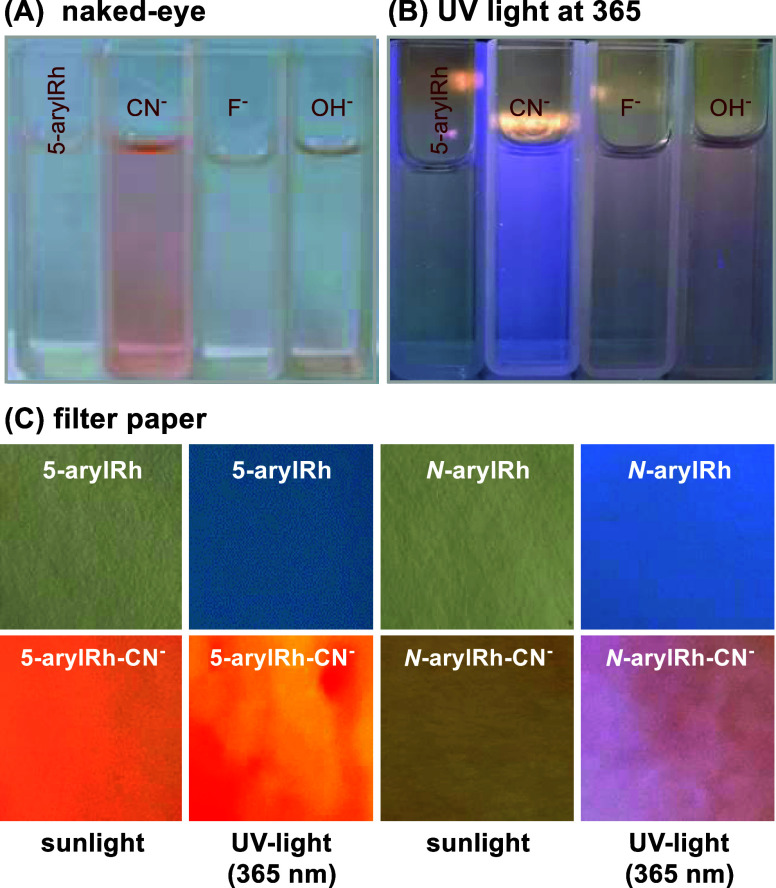
Naked-eye (A) and UV light at 365 nm (B) color changes of **5-arylRh** in the presence of selected anions in THF/H_2_O (v/v: 8:2) and (C) the photographs depicting the colorimetric response.

To evaluate the adaptability of *N*/5-arylRhs as a straightforward and efficient colorimetric and solid-state optical probe for detecting cyanide, we investigated the *N*/5-arylRhs compound as a colorimetric and solid-state probe for cyanide detection. In solution, **5-arylRh** transitioned from colorless to red upon cyanide binding, enabling naked-eye sensing (Figure 9A,B). For paper-based assays, test strips were dipped in *N*/5-arylRh solutions and exposed to [Bu_4_N]CN. These strips exhibited rapid, visible color changes under sunlight and UV light, especially with mixed [Bu_4_N]CN solutions (Figure 9C). This facile method offers a convenient and cost-effective approach for visual cyanide detection, demonstrating the potential of *N*/5-arylRhs for developing rapid and effortless cyanide sensing devices.

### 2.6 Electronic Characteristics Studies

**Figure 10:**
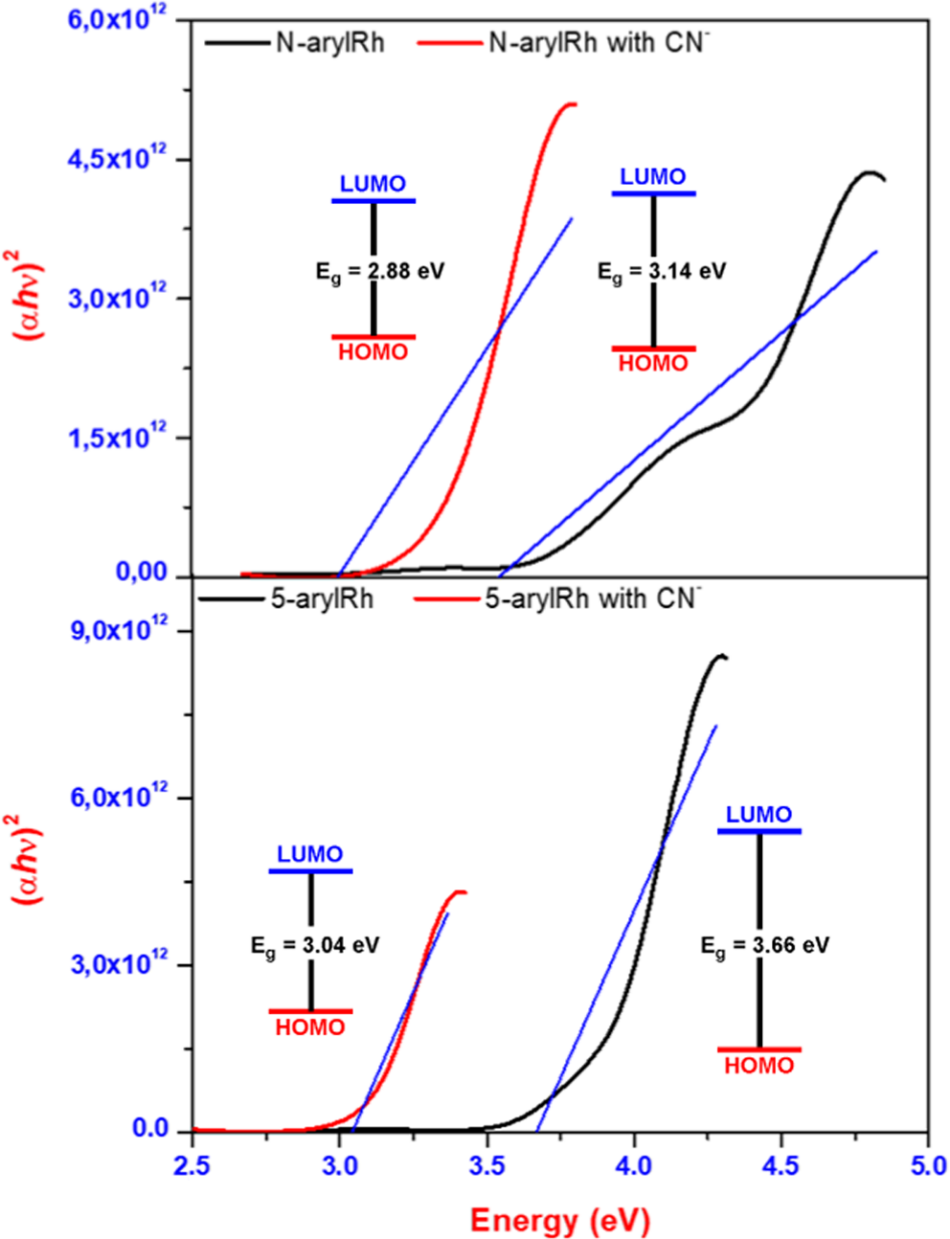
Band gap energies of **5-arylRh**/***N*-arylRh** and **5-arylRh**–CN^−^/***N*-arylRh**–CN^−^.

After conducting experimental UV–vis and fluorescence studies, the band gap energy (*E*_g_) values of the **5-arylRh**/***N*****-arylRh** and **5-arylRh**–CN^−^/***N*-arylRh**–CN^−^ complexes were determined experimentally (Figures 10 and S14). To do this, the absorption coefficient (α) was first calculated by using eq 4. Here, *d* represents the film thickness and *T* represents the percent optical transmittance value.
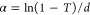
4The *E*_g_ ranges of **5-arylRh**/***N*****-arylRh** and **5-arylRh**–CN^−^/***N*-arylRh**–CN^−^ were then calculated using eq 5, where *h*ν is the photon energy and *K* is the material constant.^52^
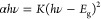
5

The *E*_g_ value of an organic molecule is correlated to its electrical conductivity and kinetic stability. An organic molecule exhibiting a wide highest occupied molecular orbital (HOMO)–least unoccupied molecular orbital (LUMO) energy gap is regarded as having heightened chemical hardness and superior stability, whereas a molecule with a narrower HOMO–LUMO energy gap is perceived as being more chemically reactive. Therefore, at this stage, to understand the nature of the fluorescence behavior, the *E*_g_ values of **5-arylRh**/***N*****-arylRh** and **5-arylRh**–CN^−^/***N*-arylRh**–CN^−^ were calculated experimentally by using absorbance values. The *E*_g_ values of **5-arylRh**/***N*****-arylRh** and **5-arylRh**–CN^−^/***N*-arylRh**–CN^−^ were found to be 3.66/3.14 and 3.04/2.88 eV, respectively. According to this, the *E*_g_ values of **5-arylRh**–CN^−^ and ***N*-arylRh**–CN^−^ are different by about 0.62/0.26 eV, which may explain why both compounds fluoresce. Additionally, the *E*_g_ value of **5-arylRh**–CN^−^ is 0.36 eV higher than that of ***N*-arylRh**–CN^−^, which may explain why **5-arylRh**–CN^−^ has a fluorescence effect.

## 3 Conclusions

In conclusion, we have successfully synthesized **5-arylRh** and ***N*****-arylRh** using an environmentally friendly approach. Subsequently, we investigated their ion detection properties. Both **5-arylRh** and ***N*-arylRh** exhibited cyanide anion-sensing capabilities as well as hydroxide and fluoride; this was also evidenced by a color change from colorless to red upon exposure to cyanide. While UV–vis studies indicated that hydroxide and fluoride ions did not interact with **5-arylRh** or ***N*****-arylRh** in the presence of increasing water ratios, fluorescence studies revealed that **5-arylRh** retained its sensing ability for cyanide and hydroxide ions under these conditions. The fluorescence and absorption spectra of the probes intensified with increasing cyanide ion concentrations. Job’s plot analysis revealed a 1:1 stoichiometry for the interaction between cyanide and both **5-arylRh** and ***N*****-arylRh**. Employing the Benesi–Hildebrand equation, the *K*_s_ values of CN^−^ to **5-arylRh** and ***N*****-arylRh** were determined to be 3.25 × 10^4^ and 7.07 × 10^4^ M^–1^, respectively. The LODs for **5-arylRh**/CN^−^, ***N*-arylRh**/CN^−^, **5-arylRh**–CN^−^/Hg^2+^, and **5-arylRh**–OH^−^/Al^3+^ were calculated as 356, 617, 414, and 1.35 μM, respectively. Furthermore, the turn-on binding mechanisms of **5-arylRh** and ***N*****-arylRh** with cyanide ions were elucidated by using relevant formulas. The experimental band gap (HOMO/LUMO) energy values obtained corroborated the proposed mechanism. Additionally, the interaction mechanism of probes with cyanide was further investigated by using the ^1^H NMR technique. Collectively, these studies suggest that **5-arylRh**, ***N*****-arylRh**, and **5-arylRh**–CN^−^ hold promise as selective and sensitive candidate sensors for CN^−^, Hg^2+^, and Al^3+^ ions.

## 4 Experimental Section

### 4.1 Synthesis of *N*/5-Monosubstituted Rhodanines

#### 4.1.1 (*E*)-3-((4-Hydroxybenzylidene)amino)-2-thioxothiazolidin-4-one (***N*-arylRh**)

A solution of 4-hydroxybenzaldehyde (**2**, 1.0 equiv) in ethanol (10 mL) was slowly added to a solution of 3-amino-2-thioxothiazolidin-4-one (**1**, 3-NH_2_–Rh, 1.0 equiv) in ethanol. The reaction mixture was stirred at room temperature for 12 h, without a catalyst. The progress of the reaction was monitored by thin-layer chromatography (TLC). After the reaction was complete, the mixture was recrystallized from ethanol to obtain the *N*-substituted rhodanine derivative ***N*****-arylRh** as a pale yellow solid (83% yield) (Scheme S1A). Mp: 263–264 °C. ^1^H NMR (400 MHz, DMSO-*d*_6_): 10.47 (s, OH, 1H), 8.50 (s, N=CH, 1H), 7.76 (d, *J* = 8.5 Hz, =CH, 2H), 6.93 (d, *J* = 8.5 Hz, =CH, 2H), 4.34 (s, CH_2_, 2H); APT ^13^C NMR (100 MHz, DMSO-*d*_6_): δ 196.8, 170.6, 169.8, 162.1, 131.2, 122.7, 116.0, 34.6 (Figure S1). The spectroscopic results are compatible with the literature.^40^

#### 4.1.2 (*Z*)-3-Amino-5-(4-hydroxybenzylidene)-2-thioxothiazolidin-4-one (**5-arylRh**)

The 3-NH_2_–Rh (**1**, 1.0 equiv) was dissolved in an EtOH/H_2_O (v/v: 9:1) solution system and boiled for about 30 min. Then, 4-hydroxybenzaldehyde (**2**, 1.0 equiv) was added dropwise to the solution. The reaction mixture was refluxed at 100 °C overnight and monitored by TLC. After the reaction was complete, the crude mixture was recrystallized from ethanol to obtain ***5*****-arylRh** as a pale yellow solid (76% yields) (Scheme S1B). ^1^H NMR (400 MHz, DMSO-*d*_6_): δ 10.52 (s, OH, 1H), 7.76 (s, =CH, 1H), 7.53 (m, A part of AB system, =CH, 2H), 6.94 (m, B part of AB system, =CH, 2H), 5.92 (bs, NH_2_, 2H); ^13^C NMR (100 MHz, DMSO-*d*_6_): δ 187.64, 164.30, 161.15, 134.65, 133.94, 124.54, 117.12, 116.05 (Figure S2). The data are compatible with the literature.^40^

### 4.2 Procedures of Photochemical Properties Measurement

To study the UV–vis and fluorescence properties of the rhodanine derivatives with various ions, we added one equivalent of each ion to a solution of rhodanine (***N*****-arylRh** or **5-arylRh**) in THF at room temperature. For the fluorescence titration study of rhodanines (***N*****-arylRh** or **5-arylRh**) with [Bu_4_N]CN, we added increasing concentrations of [Bu_4_N]CN to a solution of rhodanine (***N*****-arylRh** or **5-arylRh**) in THF. We repeated each measurement at least twice to ensure consistency. We also performed a Job’s plot measurement to determine the stoichiometry of the complex between rhodanines (***N*****-arylRh** and **5-arylRh**) and [Bu_4_N]CN. Finally, we calculated the LOD, LOQ, and association constant (*K*_s_) using standard formulas. The detailed experimental procedures are described in the Supporting Data.

## Supporting Information Available

General experimental methods and characterizations of the materials (PDF)
