# Supplementary material for: Off-On–Off Cascade Recognition of Cyanide, Mercury, and Aluminum Using N/5-Monosubstituted Rhodanines
Source: ACS Omega. 2024 Apr 4;9(15):17602–15. doi: 10.1021/acsomega.4c01066 (PMC11024942; doi:10.1021/acsomega.4c01066)
Supplement: Supplementary file 1 — ao4c01066_si_001.pdf [file ao4c01066_si_001.pdf]

Supplementary Data for

The off-on-off cascade recognition of cyanide, mercury and aluminum using *N*/5-monosubstituted-rhodanines

Sinan Bayindir[a]* and Abdullah Saleh Hussein[b]

[a]*Department of Chemistry, Faculty of Sciences and Arts, Bingöl University, Bingöl, 12000, Türkiye*

[b]*Department of Chemistry, Graduate School of Natural and Applied Sciences, Bingöl University, Bingöl, 12000, Türkiye*

Corresponding author: e-mail: sbayindir@bingol.edu.tr

Material and apparatus.

All solvents and chemicals were commercially available from Merck or Sigma-Aldrich. The reaction progress was monitored by thin-layer chromatography (TLC, 0.25-mm-thick precoated silica plates). ^{1}H and ^{13}C NMR spectras were recorded on a 400 (100)-MHz Bruker spectrometer. Infrared spectra were recorded on a Mattson 1000 FT-IR spectrophotometer. UV-Vis absorption and fluorescence spectra of samples were recorded on a Shimadzu UV-3101PL UV-Vis-NIR spectrometer and Perkin–Elmer (Model LS 55) Fluorescence Spectrophotometer, respectively.

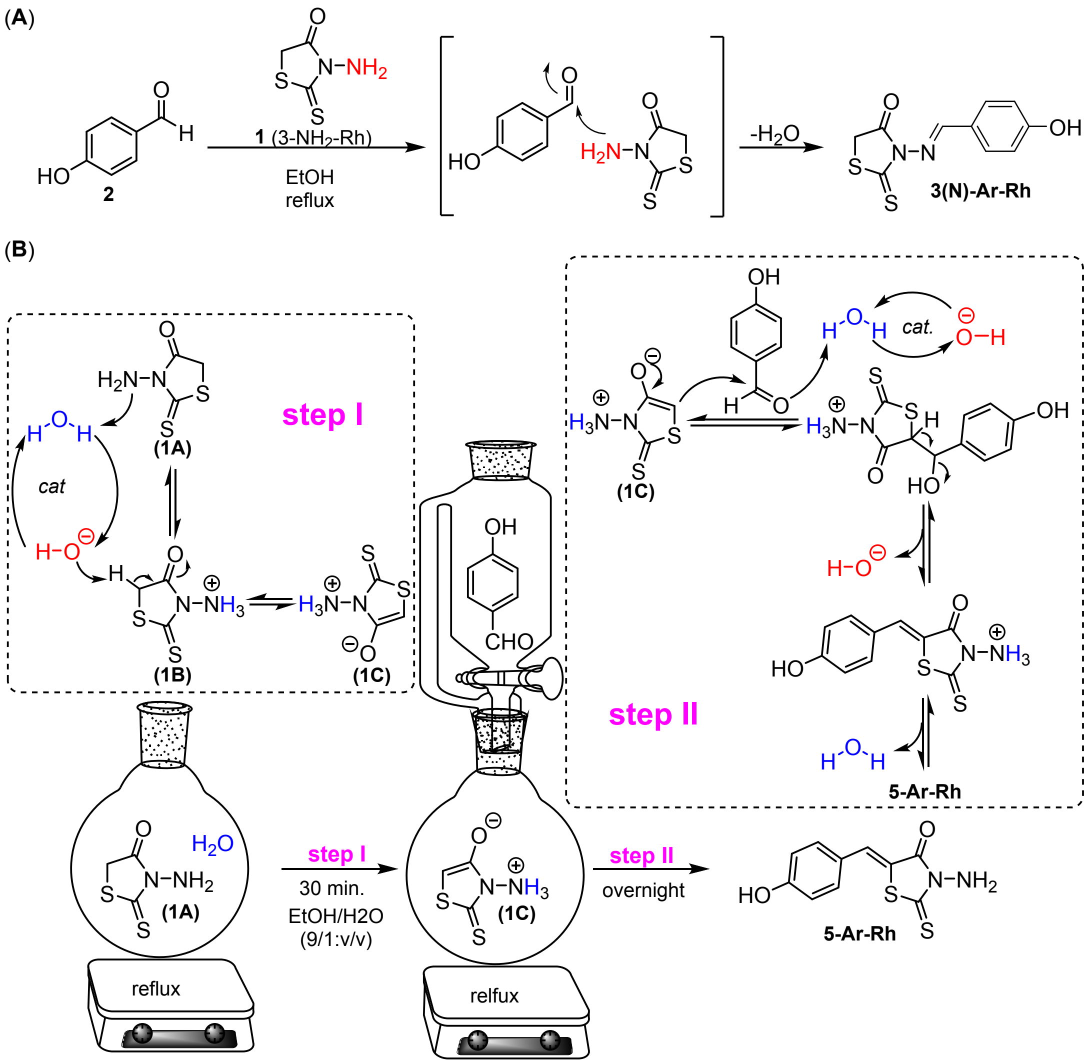

Scheme S1. The synthesis strategy of N-arylRh (**A**) / 5-arylRh (**B**), and proposed formation mechanisms

UV-Vis and fluorescence studies of N/5-arylRhs with various cations and anions: The solution of ***N*/5-arylRhs** (1×10^{-2} M) and cations (Al^{3+}, Ca^{2+}, Cd^{2+}, Co^{2+}, Cu^{2+}, Fe^{2+}, Fe^{3+}, Hg^{2+}, Mg^{2+}, Mn^{2+}, Ni^{2+}, Zn^{2+}, and Pb^{2+} as their chloride salts, 1×10^{-2} M) / anions (($[Bu_4N]F$, $[Bu_4N]Cl$, $[Bu_4N]Br$, $[Bu_4N]I$, $[Bu_4N]AcO$, $[Bu_4N]BnO$, $[Bu_4N]HSO_4$, $[Bu_4N]ClO_4$, $[Bu_4N]CN$, $[Bu_4N]SCN$, $[Bu_4N]H_2PO_4$, $[Bu_4N]OH$, 1×10^{-2} M) were prepared in THF and H_2O, respectively. A solution of ***N*/5-arylRhs** (5 μM) was placed in a quartz cell and the UV-Vis and fluorescence spectrums were recorded in THF. After introduction of the solution of cations (1 equiv.), the changes in absorbance intensity were recorded at room

temperature each time.

The fluorescence titration of N/5-arylRhs with ions: The solution of probe ***N*/5-arylRhs** (1×10^{-2} M) and ions (1×10^{-2} M) were prepared in THF and H_2O, respectively. The concentration of probe ***N*/5-arylRhs** used in the experiments was 2 µM. The fluorescence titration spectra were recorded by adding corresponding concentration of ions to a solution of ***N*/5-arylRhs** in THF or H_2O. Each titration was repeated at least twice until consistent values were obtained.

Job's plot measurement: Probe ***N*/5-arylRhs** was dissolved in THF to make the concentration of 1×10^{-2} M. 5.00, 4.50, 4.00, 3.50, 3.00, 2.50, 2.00, 1.50, 1.00, 0.50 and 0.0 mL of the ligand solution were taken and transferred to vials. Ions were dissolved in H_2O to make the concentration of 1×10^{-2} M. 0.0, 0.50, 1.00, 1.50, 2.00, 2.50, 3.00, 3.50, 4.00, 4.50, and 5 mL of the ions solution were added to each ligand solution. Each vial had a total volume of 5 mL. After shaking the vials for a few seconds, fluorescence spectra were taken at room temperature.

Determination of detection limit: The absorption and fluorescence measurements were taken for each solution containing ions. The detection limits for ions were calculated based on the absorption and fluorescence titration. For this purpose, a **3.3 SD / *slope*** equation was used. Where ***SD*** is the standard deviation of blank, the ***slope*** is the fit line in fluorescence titration experiment or in absorbance titration experiment.

LOQ = 3.3 x (Standard Deviation (SD) / Slope)

Determination of Association Constant: Association constant was calculated according to the Benesi-Hildebrand equation by fluorescence method. Association Constant $\boldsymbol{K_a}$ was calculated following the equation stated below:

$$\frac{1}{F-F_0}=\frac{1}{\left\{K_a(F_{max}-F_0)[M^{x+}]^n\right\}}+\frac{1}{F_{max}-F_0}$$

Here, $\boldsymbol{F_o}$ is the fluorescence of receptor in the absence of metal ion, ***F*** is the fluorescence recorded in the presence of added metal ion, $\boldsymbol{F_{max}}$ is fluorescence in presence of added $[M^{x+}]$ max and K_a is the association constant, where $[M^{x+}]$ is ions n is the binding stoichiometry for receptor and ions. The association constant (K_a) could be determined from the slope of the straight line of the plot of *1/F-F*$1/F\text{-}F_o$ against 1/[M].

The pH measurement: The effect of different pH environments (range of 2–12) was studied for the practical application of the probe ***N*/5-arylRhs** (10 µM, in EtOH) in the absence and presence of CN^- (30 µM, in H_2O). For this purpose, the ***N*/5-arylRhs** samples were prepared in ten different tubes, and cyanide ions dissolved in water were added. The pH values of samples were modulated by adding HCl or NaOH solution. pH values of the solution were monitored with a pH meter and/or pH stick.

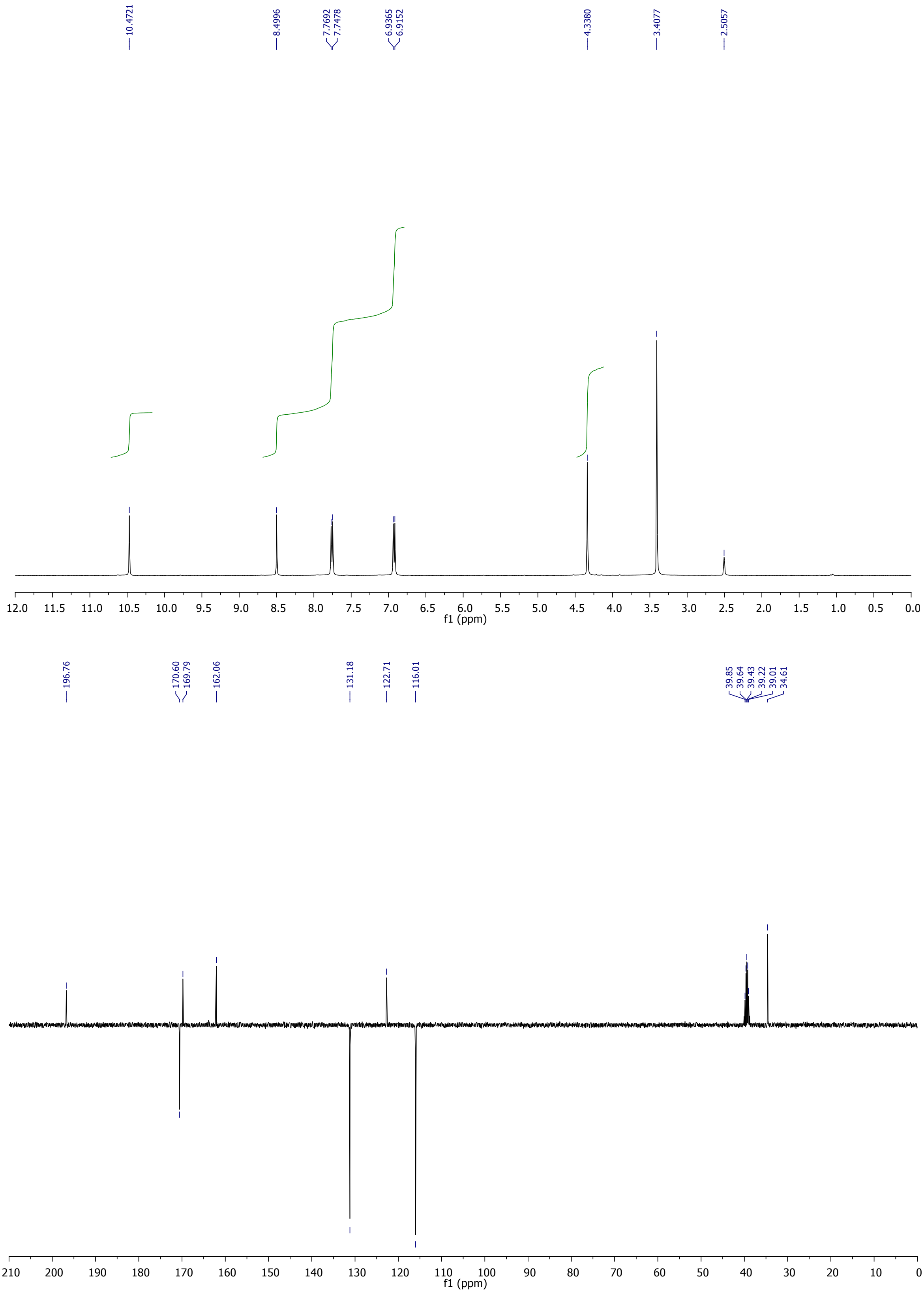

Figure S1. ^{1}H-NMR (400 MHz) and APT ^{13}C-NMR (100 MHz) spectrums of ***N*-arylRh** in DMSO-d6.

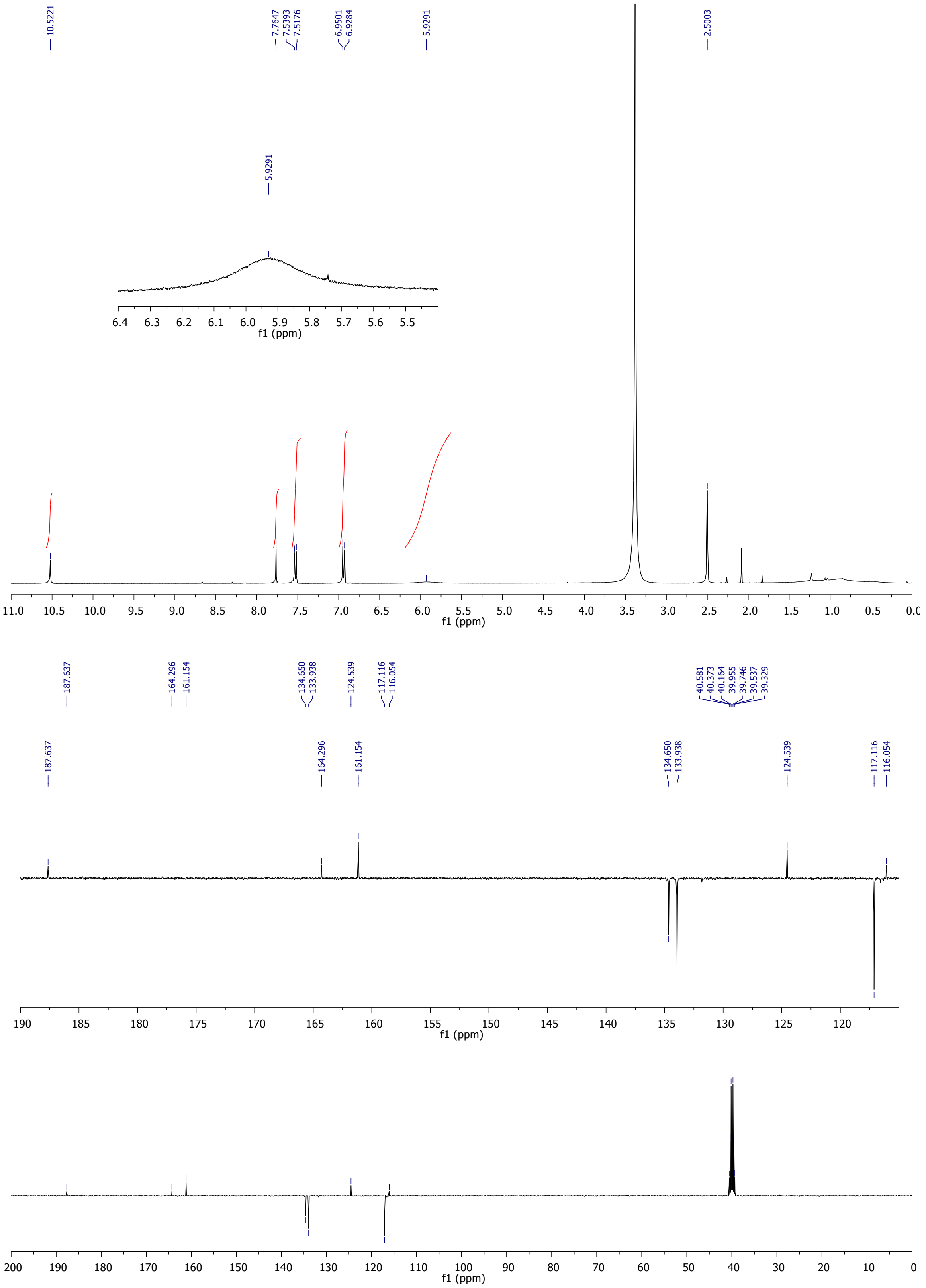

Figure S2. ^{1}H-NMR (400 MHz) and APT ^{13}C-NMR (100 MHz) spectrums of ***5*-arylRh** in DMSO-d_6.

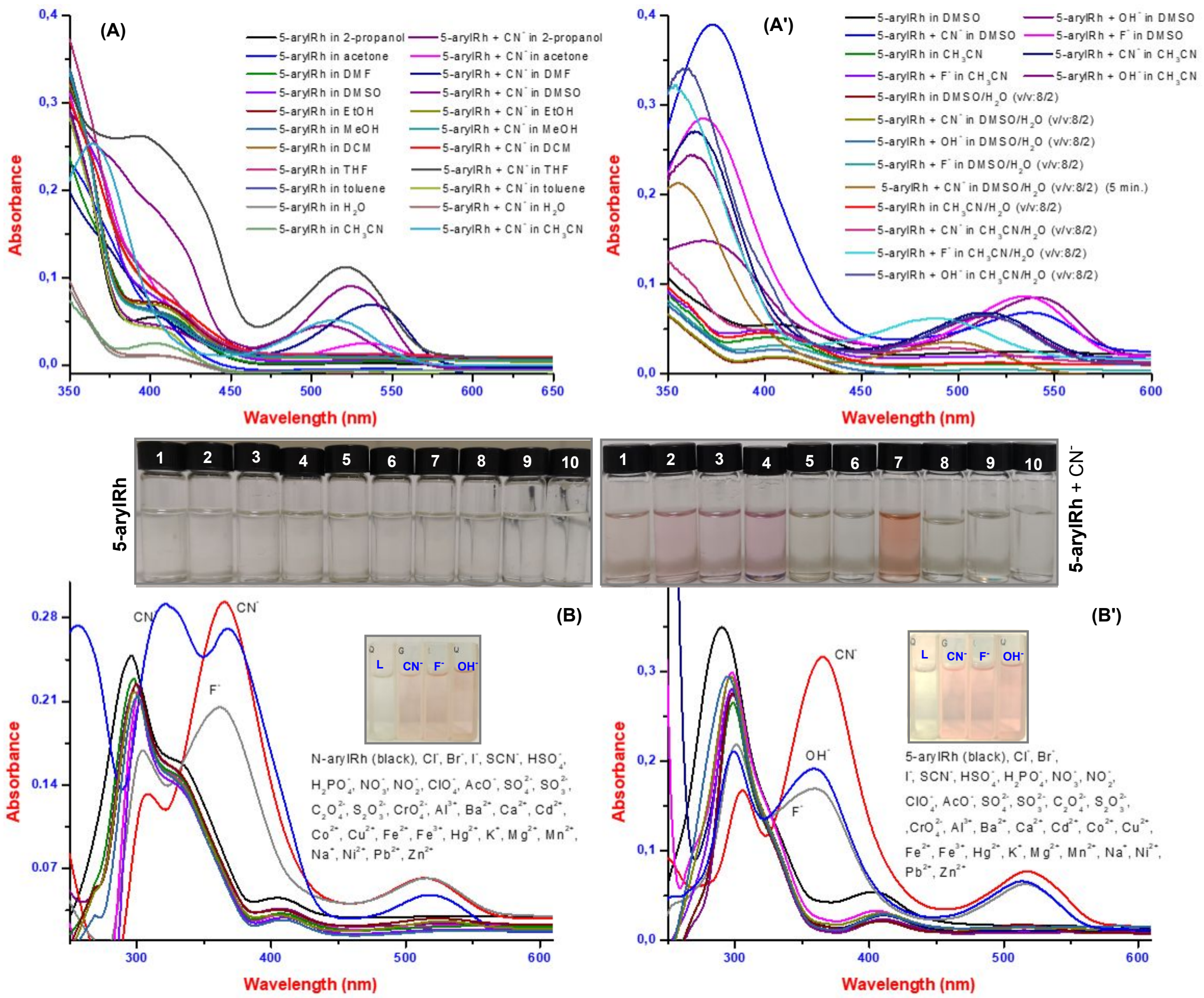

Figure S3. The UV-Vis spectrums of 5-arylRh / 5-arylRh-CN^- in different solvent systems (**A**/**A')**, and UV-Vis spectrums of 5-arylRh (**B**) and *N*-arylRh (**B'**) in THF with ions. inset pictures: colorimetric responses of Rhs with selected anions in solvent systems [(1) 2-propanol, (2) acetone, (3) DMF, (4) DMSO, (5) EtOH, (6) MeOH, (7) THF, (8) DCM, (9) Toluene, (10) H_2O]

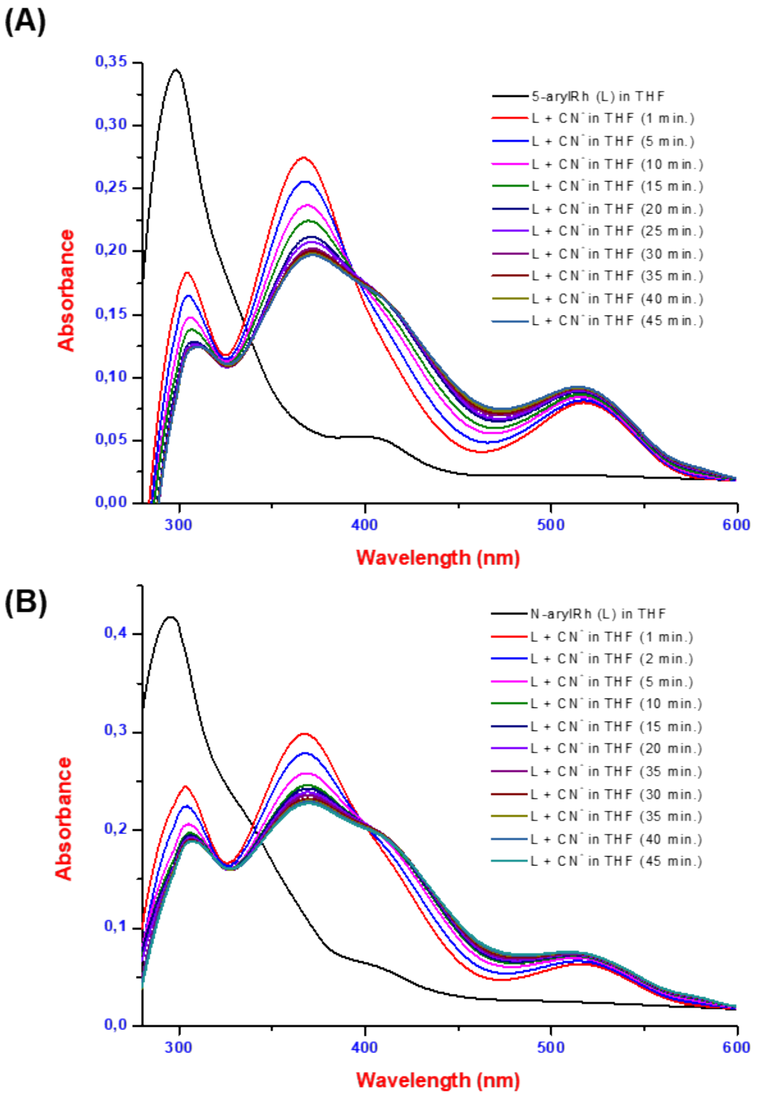

Figure S4. The UV-Vis spectrums of exposure times of 5-arylRh (**A**) and N-arylRh (**B**)

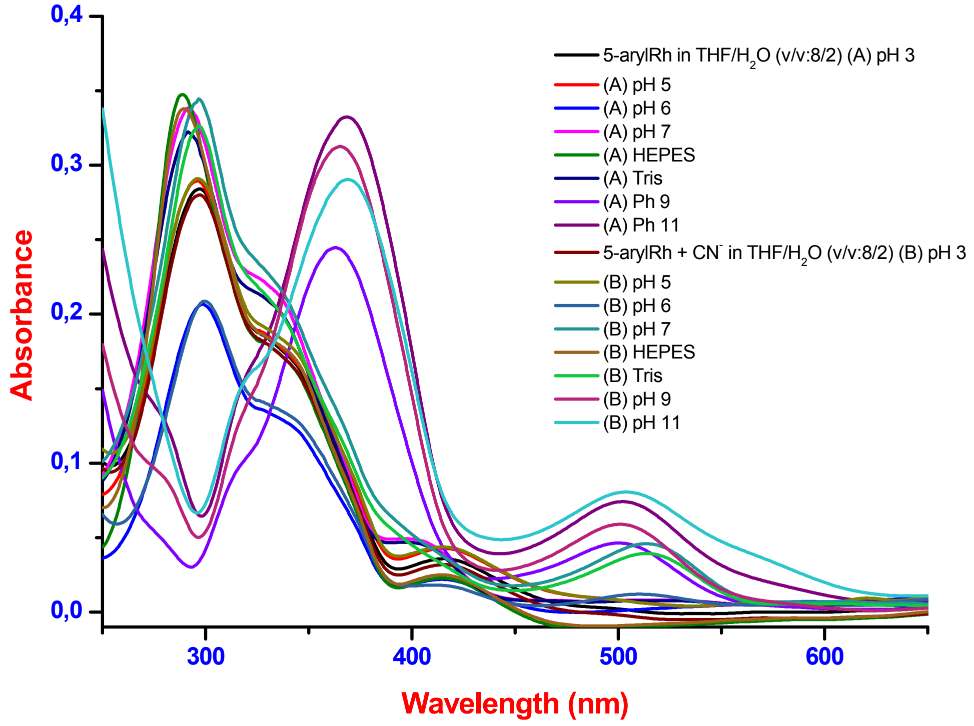

Figure S5. The UV-Vis spectra of 5-arylRh at different pH (3–11) in H_2O, the pH was modulated by adding HCl or NaOH solution.

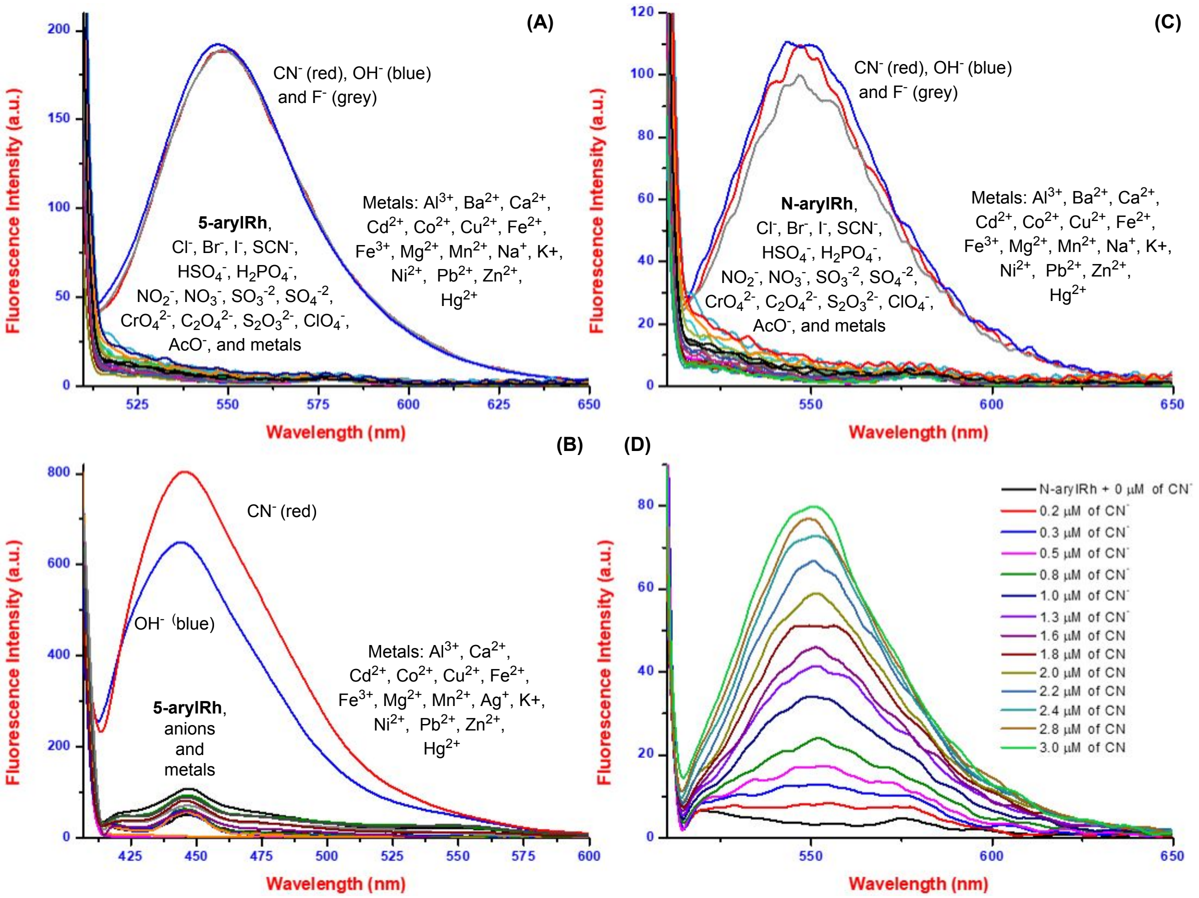

Figure S6. The fluorescence spectrums of 5-arylRh (**A** and **B**) and *N*-arylRh (**C**) with various ions, and (**D**) fluorescence titration spectra of *N*-arylRh in the presence of increasing $[Bu_4N]CN$ in THF.

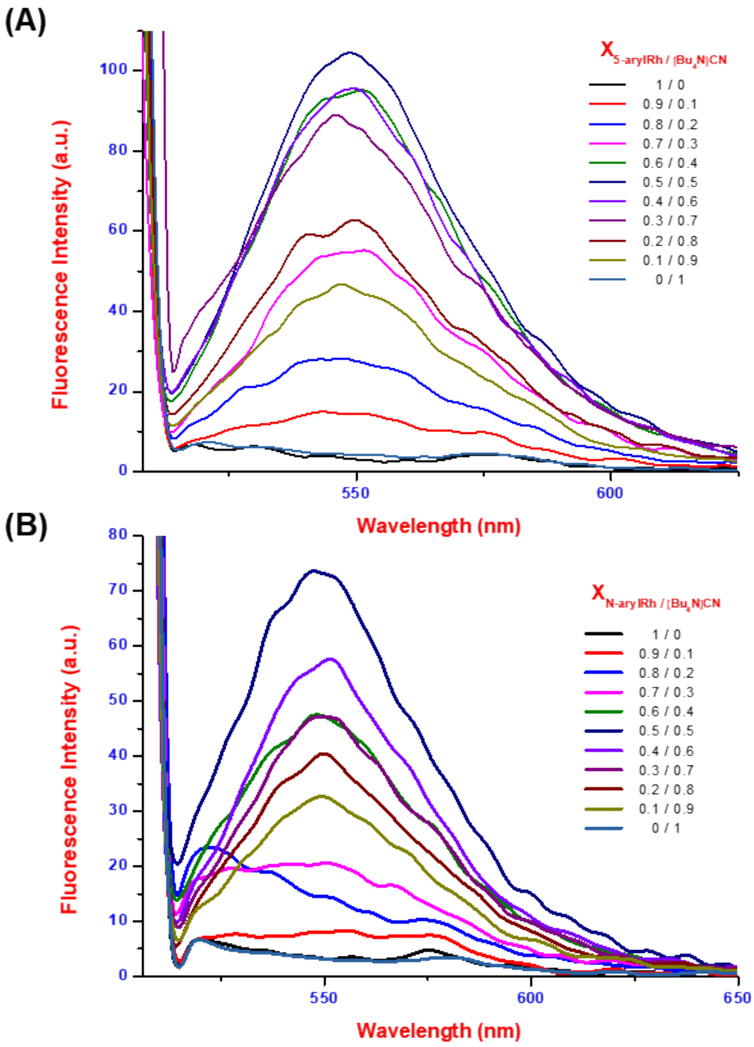

Figure S7. Job's plot fluorescence spectrums of 5-arylRh (**A**) and *N*-arylRh (**B**) with [Bu_4N]CN

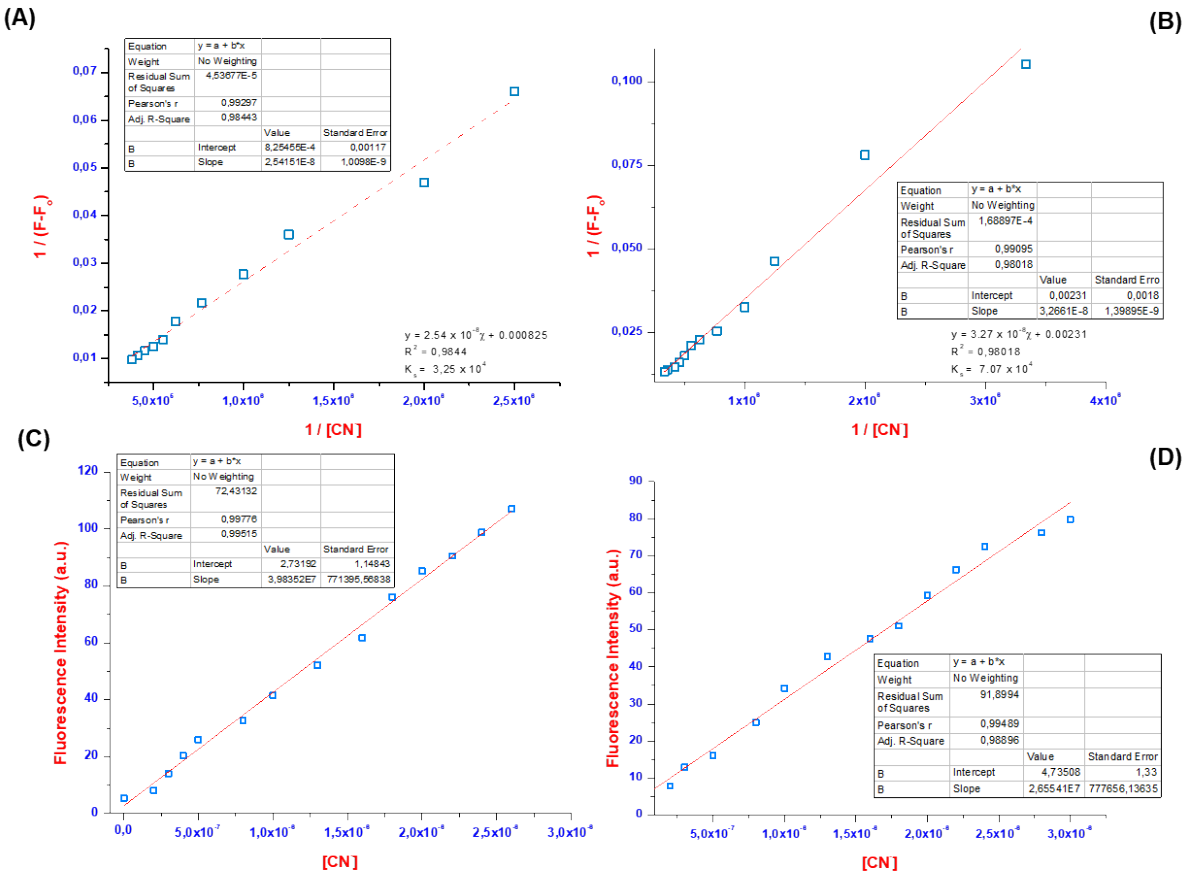

Figure S8. Benesi-Hildebrand plot based on a 1:1 association stoichiometry between 5-arylRh (**A**) or *N*-arylRh (**B**), and the change absorbance intensity of 5-arylRh (**C**) or *N*-arylRh (**D**) with the increasing concentration of [Bu_4N]CN.

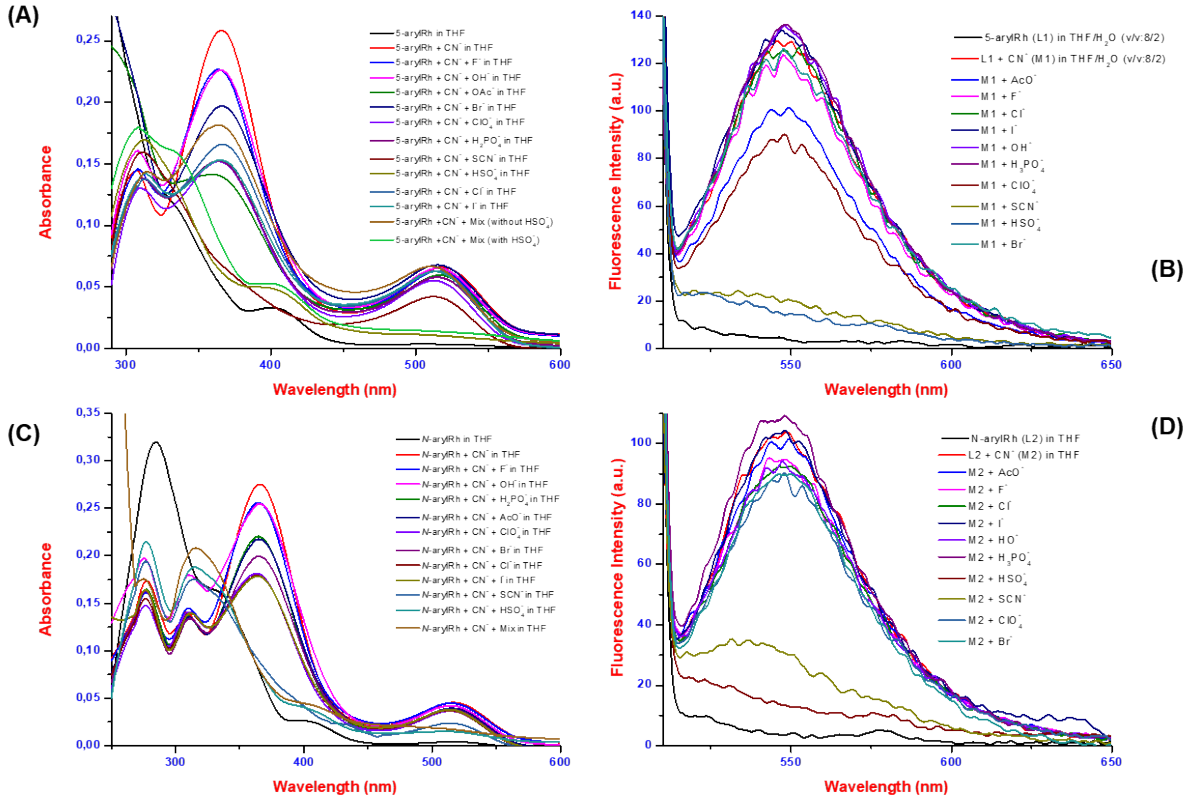

Figure S9. The UV-Vis and fluorescence spectrums of 5-arylRh-CN$^-$ (**A** and **B**) and *N*-arylRh CN$^-$ (**C** and **D**) with various anions

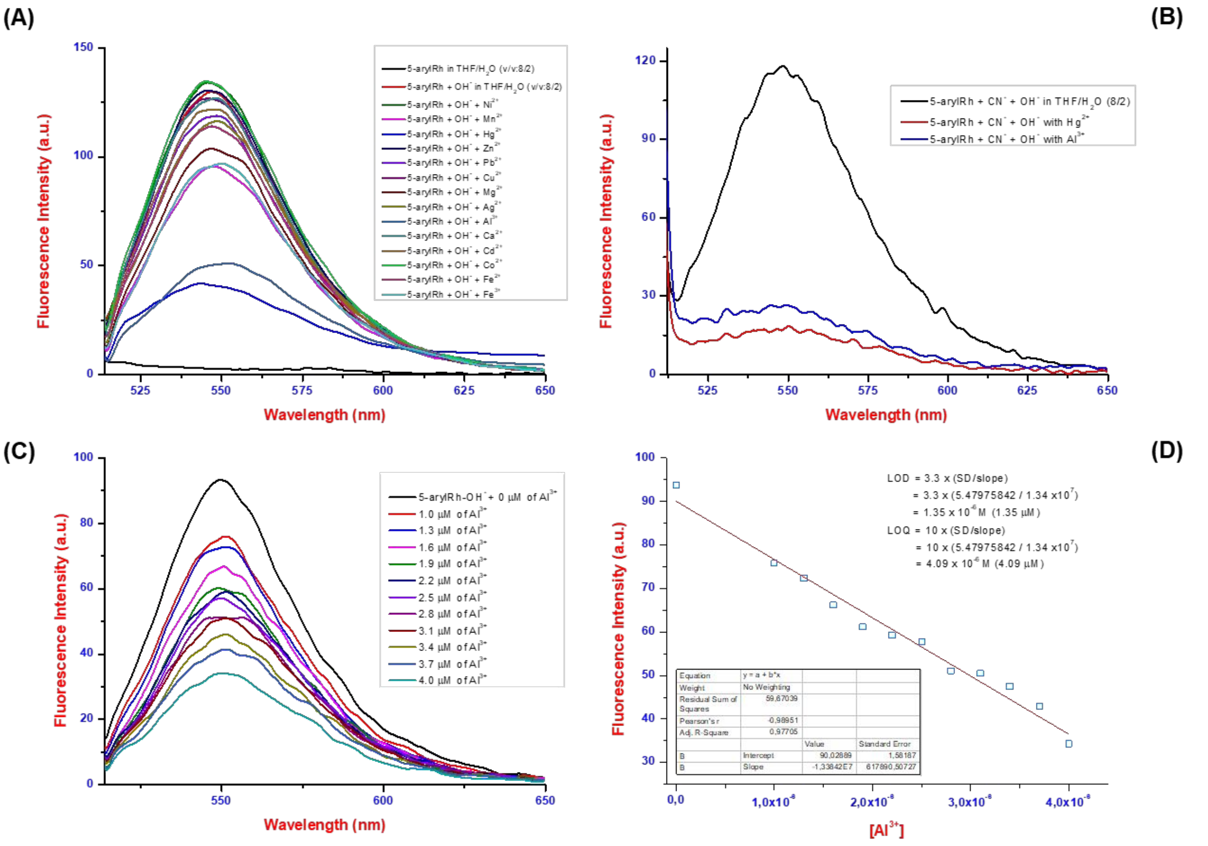

Figure S10. The fluorescence spectrums of 5-arylRh-OH¯ (**A**) and 5-arylRh-OH¯-CN¯ (**B**) with various anions, (**C**) fluorescence titration spectra of 5-arylRh-OH¯ in the presence of increasing $AlCl_3$, and (**D**) fluorescence intensity changes with increasing Al^{3+} concentration

(A)

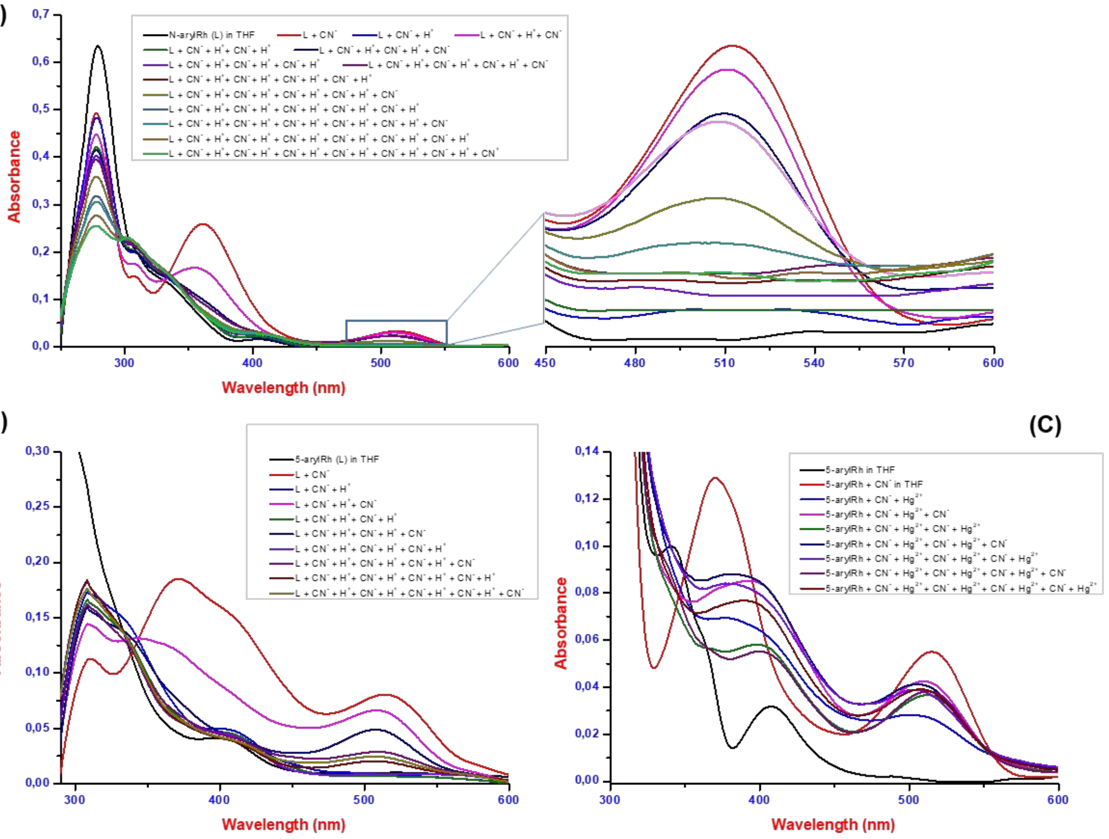

(D)

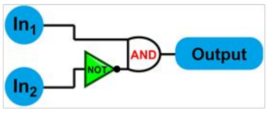

In1 (CN^-)	In2 (H^+ or Hg^{2+})	Output ($I_{514\,nm}$)
0	0	**0**
1	0	**1**
0	1	**0**
1	1	**0**

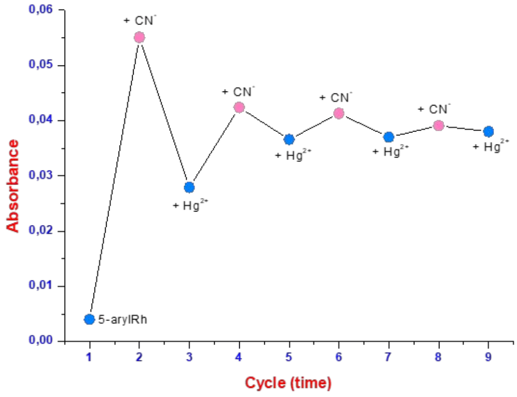

Figure S11. Reversible switching of the UV-Vis spectrum of *N*-arylRh (**A**) and 5-arylRh (**B** and **C**) upon alternate addition of CN^- and H^+ or Hg^{2+}, and (**D**) the “IMPLICATION” logic gate.

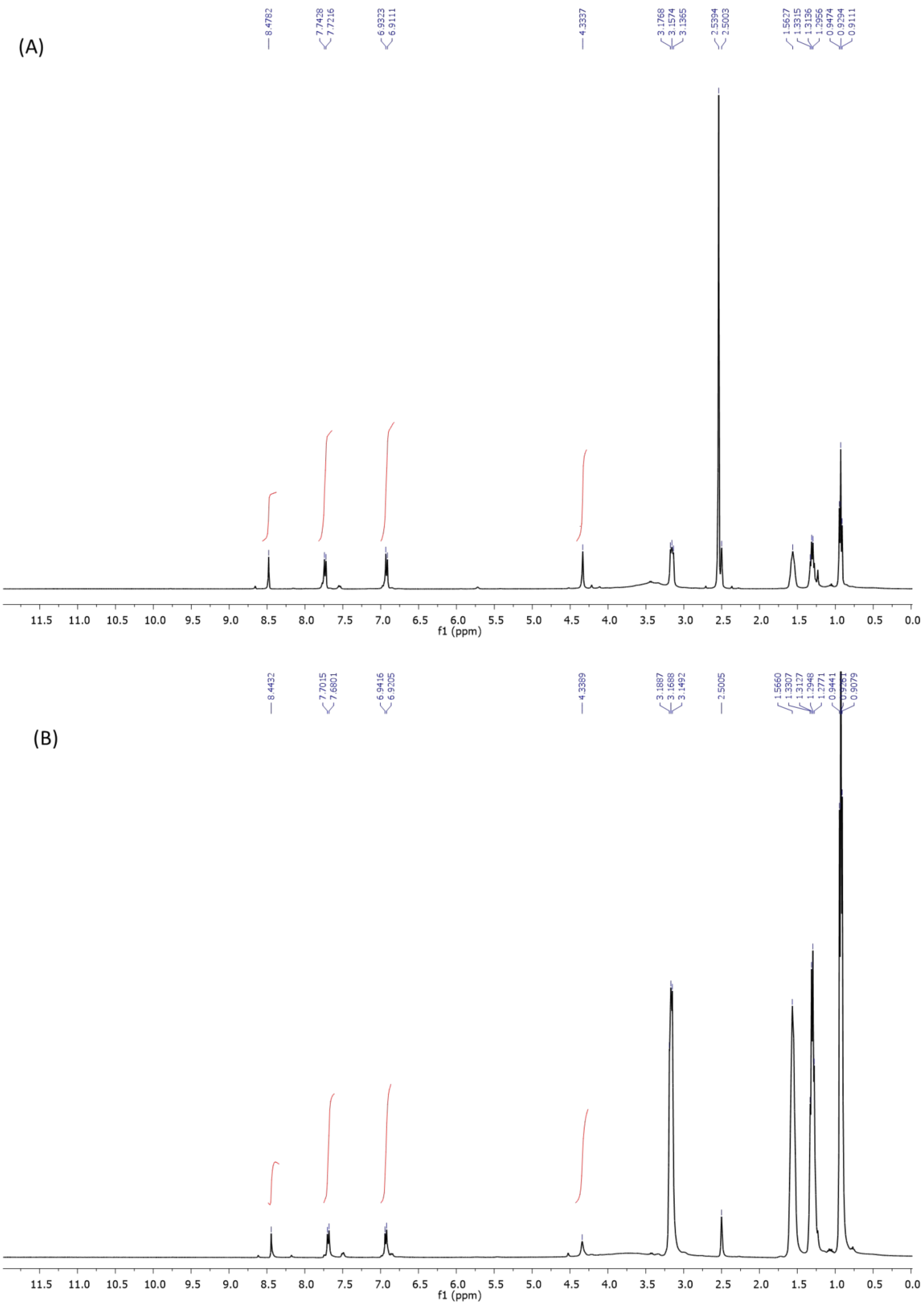

Figure S12. ^{1}H-NMR (400 MHz) spectrums of 5-arylRh+[Bu_4N]CN (**A**), and *N*-arylRh+[Bu_4N]CN (**B**) in DMSO-d6.

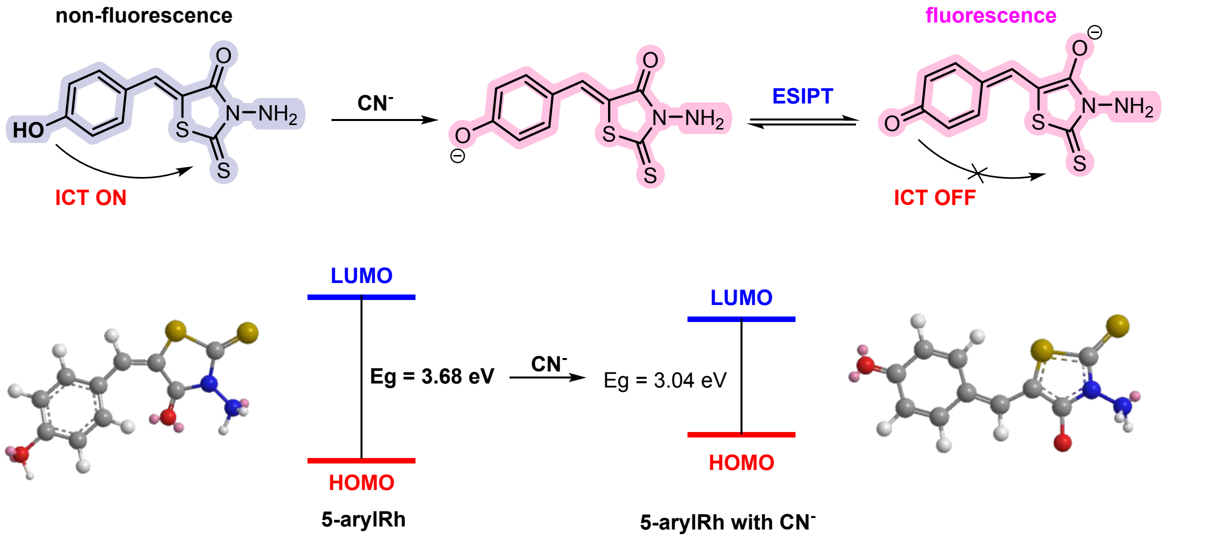

Figure S13. The proposed sensing mechanism of 5-arylRh for CN¯, the band-gap energies simulation, and the optimized structures of 5-arylRh and *N*-arylRh.

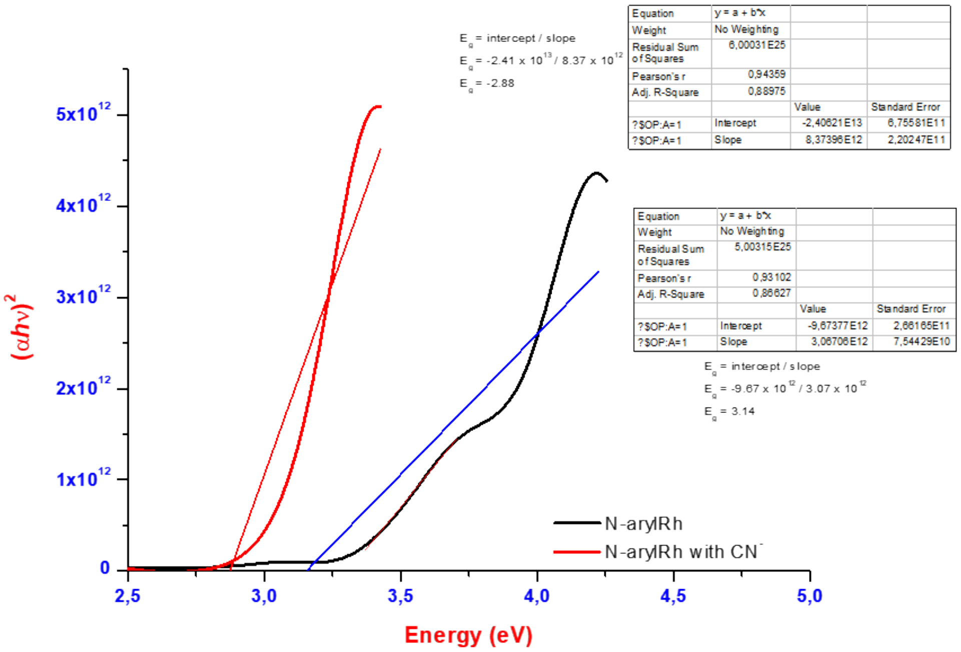

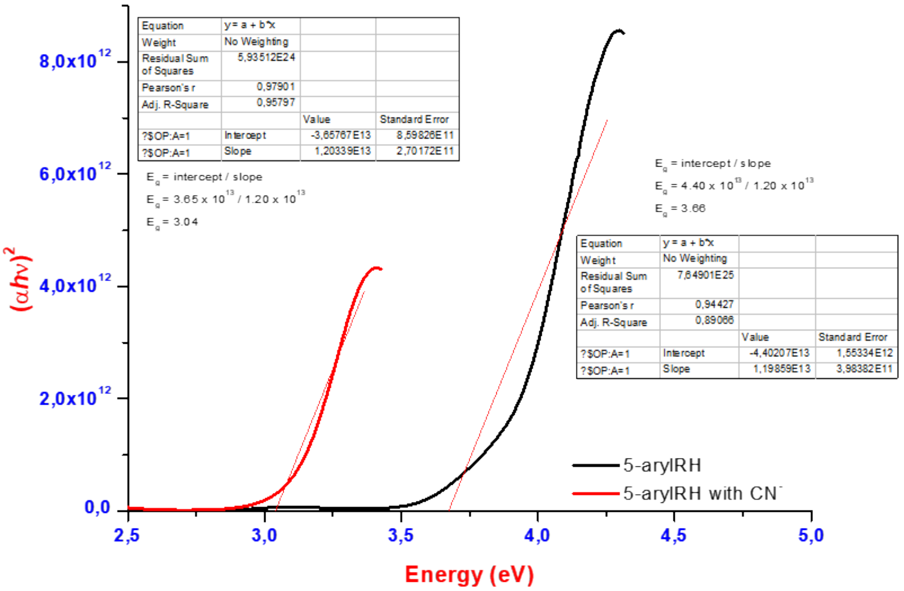

Figure S14. The band-gap energies details of 5-arylRh / *N*-arylRh and 5-arylRh-CN^{-} / *N*-arylRh-CN^{-}
